# Development of a Ratiometric Fluorescent Cu(II) Indicator Based on Poly(*N*-isopropylacrylamide) Thermal Phase Transition and an Aminopyridyl Cu(II) Ligand

**DOI:** 10.3390/molecules28207097

**Published:** 2023-10-15

**Authors:** Lea Nyiranshuti, Emily R. Andrews, Leonid I. Povolotskiy, Frances M. Gomez, Nathan R. Bartlett, Arun Timothy Royappa, Arnold L. Rheingold, William Rudolf Seitz, Roy P. Planalp

**Affiliations:** 1 Department of Chemistry, University of New Hampshire, Durham, NH 03824, USA; leah.nshuti@gmail.com (L.N.); era1004@unh.edu (E.R.A.); leopov123@gmail.com (L.I.P.); fms1020@usnh.edu (F.M.G.); nathan.r.bartlett@unh.edu (N.R.B.);; 2 Department of Chemistry, University of West Florida, Pensacola, FL 32514, USA; royappa@uwf.edu; 3 Department of Chemistry, University of California San Diego, La Jolla, CA 92093, USA; arheingold@ucsd.edu

**Keywords:** ratiometric sensing, copper, zinc, pyridyl ligands, formation constant

## Abstract

An aqueous Cu^2+^ and Zn^2+^ indicator is reported based on copolymerizing aminopyridine ligands and the environment-sensitive dansyl fluorophore into the responsive polymer poly(N-isopropylacrylamide) (PNIPAm). The metal ion binding creates charge and solvation that triggers PNIPAm’s thermal phase transition from hydrophobic globule to hydrophilic open coil. As a basis for sensing the metal-binding, the dansyl fluorescence emission spectra provide a signal at ca. 530 nm and a signal at 500 nm for the hydrophobic and hydrophilic environment, respectively, that are ratiometrically interpreted. The synthesis of the title pyridylethyl-pyridylmethyl-amine ligand (acronym PEPMA) with a 3-carbon linker to the copolymerizable group, aminopropylacrylamide (PEPMA-C3-acrylamide), is reported, along with a nonpolymerizable model ligand derivative. The response of the polymer is validated by increasing temperature from 25 °C to 49 °C, which causes a shift in maximum emission wavelength from 536 nm to 505 nm, along with an increase in the ratio of emission intensity of 505 nm/536 nm from 0.77 to 1.22 (λ_ex_ = 330 nm) as the polymer releases water. The addition of divalent Cu or Zn to the indicator resulted in a dansyl emission shift of 10 nm to a longer wavelength, accompanied by fluorescence quenching in the case of Cu^2+^. The addition of EDTA to the Cu^2+^-loaded indicator reversed the fluorescence shift at 25 °C to 35 °C. The affinities of Cu^2+^ and Zn^2+^ for the PEPMA derivatives are log *K*_f_ = 11.85 and log *K*_f_ = 5.67, respectively, as determined by potentiometric titration. The single-crystal X-ray structure of the Cu^2+^-PEPMA derivative is five-coordinate, of-geometry intermediate between square-pyramidal and trigonal-bipyramidal, and is comparable to that of Cu^2+^ complexes with similar formation constants.

## 1. Introduction

Water contamination due to industrial and agricultural development is a growing problem. Framed in terms of planetary boundaries [1], freshwater use is presently at a level of ca. 2600 km^3^ year^–1^, which is below an estimated boundary of 4000 km^3^ year^–1^; however, freshwater use is exceeding local boundaries at parts of the planet in North America, Asia, and Europe [1]. Contaminants that threaten the freshwater supply include aqueous copper, for which the US Environmental Protection Agency’s accepted maximum level in drinking water is 1.3 mg/L [2], and in wastewater effluents, no more than 6.5 mg/L [3]. Cu^2+^ in amounts of 10–100 µg/L are essential for life [4,5]; however, amounts over the EPA limits cause human illness, and the death of aquatic organisms [6]. It is therefore important to develop methods to monitor Cu^2+^ in natural waters. 

**Figure 1:**
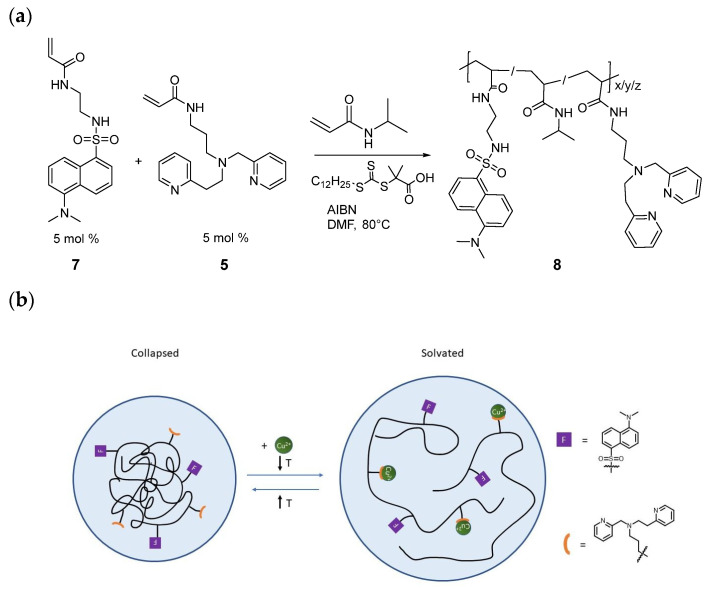
(**a**) Synthesis of the indicator **8** from PNIPAm, PEPMA-C3-acryamide (**7**), and dansyl-en-acrylamide (**5**). (**b**) Pictorial representation of the sensor design with change in temperature and addition of metal ion: ligand (orange), fluorophore (purple), polymer backbone (black), and metal ion (green).

To detect low copper concentrations from small samples, fluorescent indicators are most desirable [7]. However, Cu^2+^ is a paramagnetic ion that quenches fluorescence when bound near a fluorophore [8,9]. The mode of detection is therefore a loss of fluorescence upon binding, or “turn-off” response [10,11]. A turn-off response cannot be distinguished from other processes that diminish fluorescence, such as indicator degradation or photobleaching. We have approached the challenges of paramagnetic ion sensing by designing ratiometric fluorescent Cu^2+^ indicators that are based on polymer phase transitions monitored by a fluorophore that is remote from the Cu^2+^-binding site [12,13,14,15]. The indicator structure and its synthesis is shown in Figure 1a, and its mode of action in Figure 1b. Poly(N-isopropylacrylamide) (PNIPAm) is soluble in water below its LCST due to the hydrogen bonding between water and the amide group, and it exists in a coiled form. Hydrogen bonds are broken at the lower critical solution temperature (LCST) of 32 °C, and the polymer precipitates out of solution as a globule form. Complexation of a metal ion to the polymer at a temperature above LCST makes the polymeric environment more solvated, which drives the polymer to the soluble coiled form. The environment-sensitive fluorophore dansyl provides a readout of polymer coil/globule status indicated by a fluorescence shift. As dansyl is subjected to the more hydrophilic coil environment, emission intensity increases and maximum emission wavelength decreases, which is due to an increase in hydrogen bonding to the dimethylanilino group of dansyl. 

**Figure 2:**
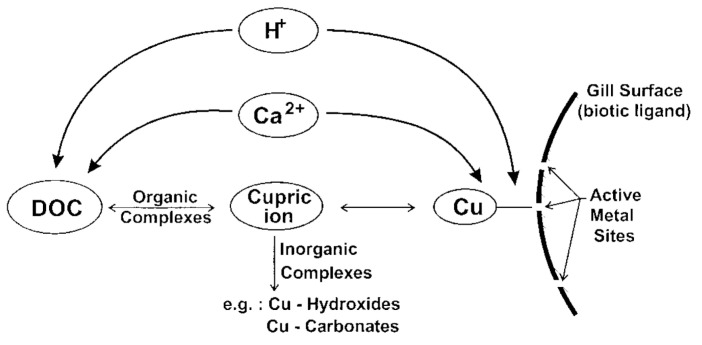
The biotic ligand model (BLM) illustrating metal complexation on chelants (DOC = dissolved organic carbon) and on the gill surface, referred to as a biotic ligand.

Through the design of appropriate Cu^2+^ ligands, incorporated in the structure of Figure 1a, we seek to detect specific ranges of Cu^2+^ concentration. The range to be monitored is determined by the concentration of aqueous, unligated Cu^2+^, and is prescribed by Cu^2+^ speciation in natural waters and the uptake of copper by aquatic organisms [16]. Current theories of environmental copper toxicity focus on its bioavailability, referred to as the biotic ligand model (Figure 2). The copper in natural waters is usually bound to natural aqueous ligands, particularly to the carboxylate groups of humic and fulvic acid. Referred to as “dissolved organic carbon” (DOC), these acids’ affinity for Cu^2+^ yields an approximate concentration of free Cu^2+^ in the 100 nM range. In the example of toxicity to fish species, copper binds to carboxylate groups of membrane glycolipids, which thereby fatally disrupts the Na^+^ ion regulatory process [17,18,19]. The toxicity of copper is therefore a question of competition for copper between the natural ligands in water and the ligands of the organism, both of which contain carboxylate ligands. 

**Scheme 1:**
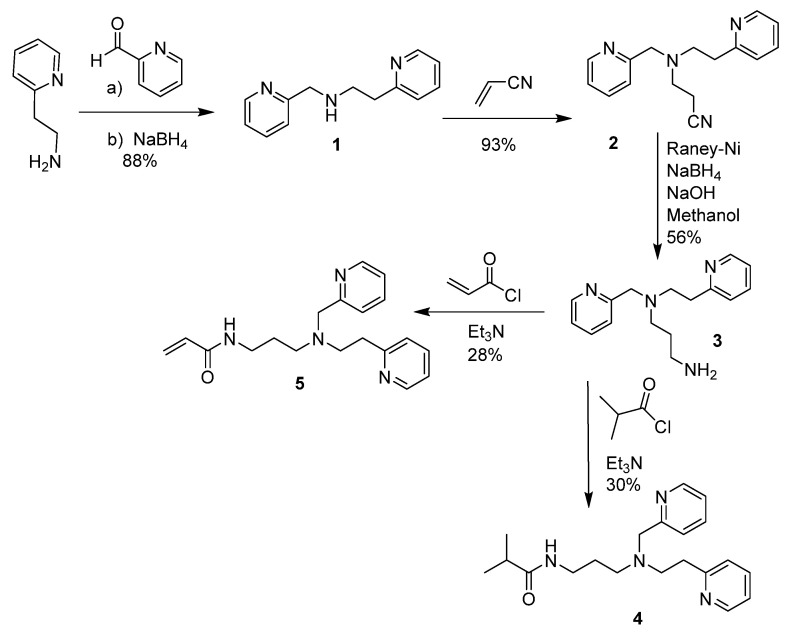
Synthesis of PEPMA-C3-acrylamide **5** and PEPMA-C3-model ligand **4**. Step (a): 2-pyridinecarboxaldehyde, methanol, 2 h; step (b): NaBH_4_, methanol, 24 h.

Herein, we report indicator development using the ligand (PEPMA-C3-acrylamide) (Scheme 1) and the methodology of Figure 1b. The design of the base Cu^2+^–ligand, PEPMA, was inspired by the desire to create an asymmetric bonding environment for Cu^2+^. PEPMA binds Cu^2+^ providing two different ring sizes, one five-membered and one six-membered, which can accommodate the differences in Cu^2+^–ligand bond lengths in accord with the Jahn–Teller effect. 

## 2. Results and Discussion

### 2.1. Synthesis and Characterization of PEPMA-C3-Acrylamide, the PEPMA-C3-Isobutyramide Model Ligand, and the Indicator Polymer **8**

In order to use PEPMA in a polymeric indicator, it was necessary to prepare PEPMA-C3-acrylamide **5.** Acrylamide **5** was synthesized via condensation of 2-(2-ethylamino) pyridine and 2-pyridinecarboxyaldehyde followed by in situ reduction with NaBH_4_ to obtain the corresponding secondary amine **1** (Scheme 1). Because the attachment of the coordinating N to the carbonyl of the acryloyl group would reduce its basicity and therefore the ligand affinity for Cu^2+^, a three-carbon spacer unit was incorporated between the metal-coordinating motif and the acrylamide group. Michael addition between **1** and acrylonitrile afforded **2,** which was then reduced to primary amine **3** with Raney-Ni and NaBH_4_. The addition of isobutyryl chloride or acryloyl chloride to **3** afforded the PEPMA-C3-isobutyramide model ligand **4** or the PEPMA-C3-acrylamide ligand **5**, respectively. Ligand **4** was prepared for structural studies because the acryloyl group present in **5** would be unstable during metal complexation studies. The Cu^2+^-complexation properties of PEPMA in ligand **4** were verified by a Job’s plot study, which indicated maximum absorbance at approximately 1:1 metal:ligand ratio (Supplementary Materials). 

To incorporate the fluorescent group dansyl into the indicator, a polymerizable derivative was prepared by the addition of ethylenediamine to dansyl chloride, followed by amidation with acryloyl chloride to afford **7**. Copolymerization of ligand **5** and fluorophore **7** with N-isopropylacrylamide afforded the target metal ion indicator **8** (Figure 1a). The indicator was prepared with 4.3 mol % of PEPMA-C3-acrylamide and 2.35 mol % of the dansyl fluorophore, as informed by ^1^H NMR (Supplementary Materials). Monomer incorporation percentages of PEPMA ligand **5** and dansyl fluorophore **7** were determined as 4.1% and 2.1%, respectively, as determined with ^1^H-NMR (Supplemental Materials). An average molecular weight of 34,000 g/mol was determined via diffusion order spectroscopy (DOSY) NMR (Supplementary Materials) [19].

### 2.2. Structural Studies

**Figure 3:**
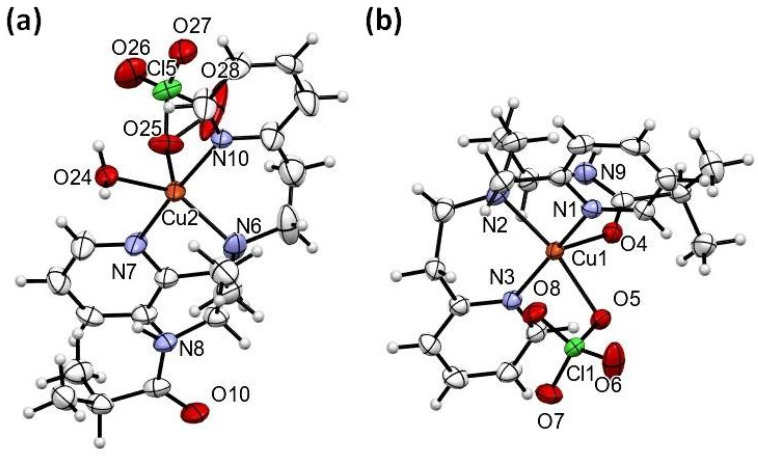
ORTEP plot (50% probability) for (**a**,**b**).

To determine bonding modes of the PEPMA-C3-amide motif with Cu^2+^, a complex was formed between the PEPMA model ligand **4** and Cu(ClO_4_)_2_, isolated as single crystals, and subjected to single-crystal X-ray crystallographic study. We observe two independent complexes, (**a**) and (**b**), of Cu bound to **4** and to one ClO_4_ counterion in the asymmetric unit (Figure 3). In both complexes, a distorted trigonal-bipyramidal coordination geometry obtains. In form **a,** Cu^2+^ is coordinated to the three nitrogen donor atoms of PEPMA with bond distances Cu2-N6 = 2.037(5) Å, Cu2-N7 = 1.991(4) Å, and Cu2-N10 = 1.979(3) Å. Copper is also coordinated to water and one perchlorate molecule, where the bond lengths for Cu2-O24 and Cu2-O25 are 2.055(3) Å and 2.128(6), respectively. Form **b** is also coordinated to the three nitrogen atoms from the PEPMA-C3-model ligand, but contrasts to **a** because the oxygen of the amide group, O4, is coordinated to copper in place of water. The bond lengths for Cu1-N1, Cu1-N2, Cu2-N3, Cu1-O4, and Cu1-O5 are 1.962(3) Å, 2.042(4) Å, 1.956(3) Å, 2.097(3) Å, and 2.164(3) Å, respectively. 

Interestingly, the coordination sphere of form **b** reveals the flexibility of the three-carbon spacer unit that can allow coordination of the amide oxygen, which may also occur in the polymeric indicator **8**. Flexibility in the coordination sphere of form **a** is also seen in the disorder of oxygens in the coordinated perchlorate anion. In other aspects, the distortions from the ideal trigonal-bipyramid are seen in both the **a** and **b** forms of [Cu(**8**)(ClO_4_)]^+^, marked by the angles N10-Cu2-N7 = 171.9(2)° and N6-Cu2-N7 = 83.8(2)° in **a** compared with N1-Cu1-N2 = 82.6(1)° and N1-Cu1-N3 = 168.3(1)° in **b**, similarly distorted from the values of 180° and 90°. These are typical behaviors of Cu^2+^ coordination complexes [20].

### 2.3. Formation Constants of PEPMA-C3-Isobutyramide (4) with Cu^2+^ and Zn^2+^

**Figure 4:**
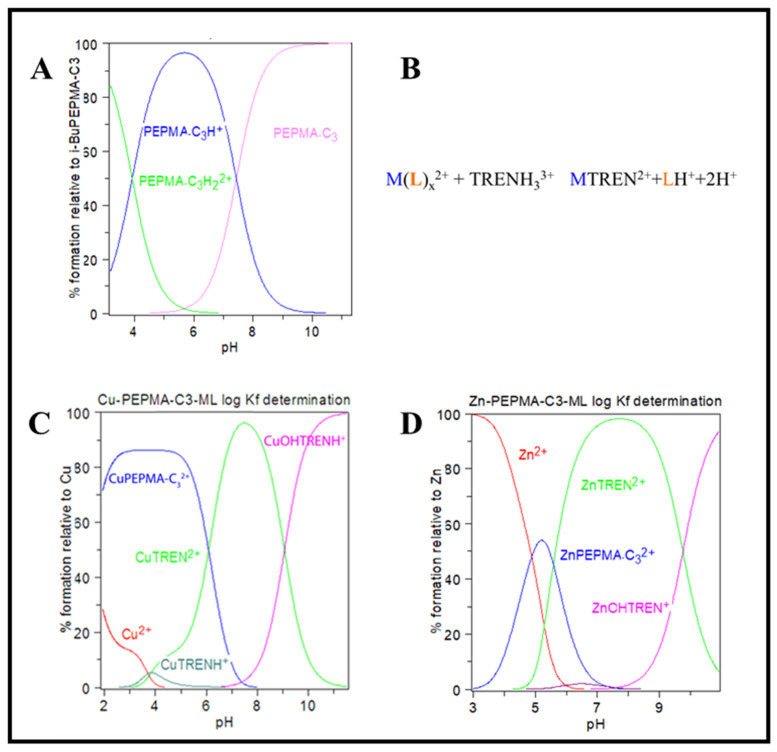
Speciation plots for formation constant determinations of the PEPMA-C3 model ligand **4** with *I* = 0.1 NaNO_3_ at 25 °C. (**A**): p*K*a determination of the PEPMA-C3-model ligand **4** (here denoted as PEPMA-C_3_ or i-BuPEPMA-C_3_) with 2 mol equiv. of HNO_3_. [ML] = 1.86 mM and *I* = 0.1 NaNO_3_ at 25 °C. (**B**) Equation for formation constant determination; here, **4**, denoted as L. TREN, is used as a competing ligand: [M(L)_x_]^2+^ + [TRENH_3_]^3+^. Ý [M(TREN)]^2+^ + LH^+^ + 2H^+^. (**C**): Distribution diagram of the PEPMA-C3-model ligand **4** with Cu^2+^ and TREN species. [Cu^2+^] = [4] = [TREN•3HCl] = 1.86 mM. (**D**): Distribution diagram of PEPMA-C3 model ligand **4** with Zn^2+^ and TREN species. [Zn^2+^] = [4]= [TREN•3HCl] = 1.86 mM.

To estimate the working concentration of indicator **8** for Cu^2+^ and its selectivity for Cu^2+^ vs. the other transition metal dication in wastewater, Zn^2+^, we measured formation constants of **4** for Cu^2+^ and Zn^2+^. Potentiometric titrations to measure pKas and formation constants were performed on the PEPMA-C3-isobutyramide **4** rather than **5** because it more closely resembles the ligand substituents once polymerized. Because this technique is based on the competition of H^+^(aq) for M^2+^, pKas were measured first. The p*K*_a1_ and p*K*_a2_ of **4** were 3.84 and 7.45, respectively (Figure 4A). Because of the weak basicity (p*K*_a1_ = 3.84) of **4** and its relatively high affinity for Cu^2+^, **4** makes a complex with Cu^2+^ as soon their solutions are mixed. To allow for competition between protons and metal ions, the more basic ligand tris(2-aminoethyl) amine (TREN) was used as a competing Cu^2+^ ligand for the potentiometric titrations (Figure 4B) [21]. 

Distribution analysis revealed that Cu(PEPMA-C_3_)^2+^ is the dominant species at acidic pH (below pH 6), followed by formation of Cu(TREN)^2+^ and copper hydroxide species at higher pH values (Figure 4C). The data were fitted via least squares analysis to afford a Cu^2+^ affinity of log Kf = 11.65. To assess competition between Zn^2+^ and Cu^2+^ for the indicator ligand, we performed a potentiometric titration to determine the formation constant of **4** for Zn^2+^. The speciation diagram (Figure 4D) of Zn^2+^ and **4** showed that free Zn^2+^ ions are the dominant species at acidic pHs. Increasing pH resulted in the formation of [Zn(L)]^2+^ with a log Kf of 5.67 at approximately pH 6, followed by formation of Zn(TREN)^2+^ and ZnOHTREN^+^ at higher pH. These studies showed that **4** is selective for Cu^2+^ relative to Zn^2+^ in accordance with the Irving–Williams series [22]. 

### 2.4. Thermally Dependent Fluorescence of Indicator **8** Alone and in Presence of Metal Ion

**Figure 5:**
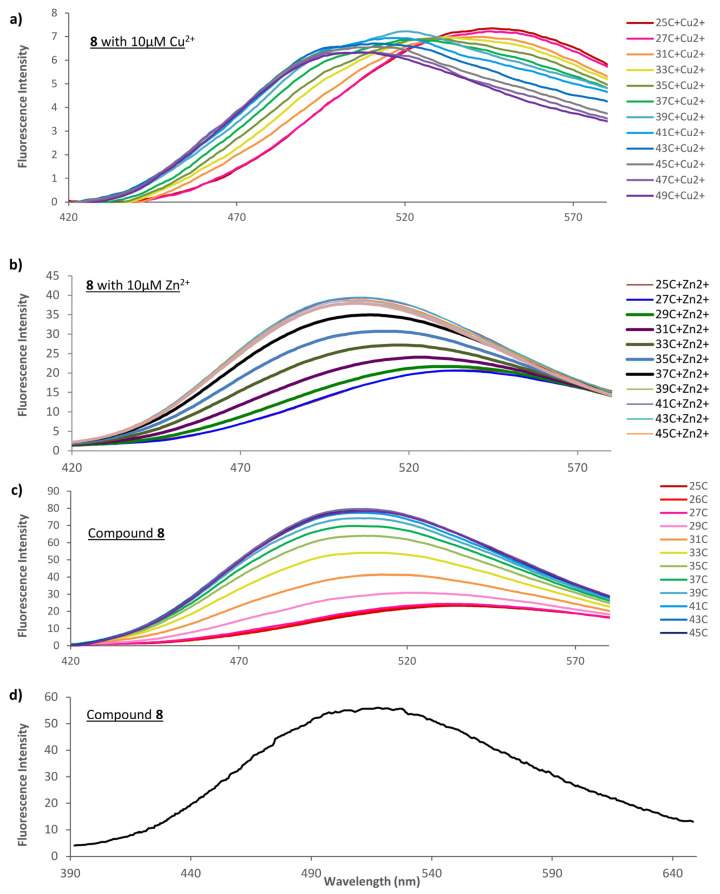
Fluorescence spectra of the indicator **8** (0.1 M MES buffer, pH 6.0) as a function of increasing temperature. λ_ex_ = 330 nm; excitation slit width = 10; and emission width = 10. (**a**) Addition of 0.01 mM of Cu^2+^ to **8**, 0.005 g/L; (**b**) addition of 0.01 mM of Zn^2+^ to **8**, 0.005 g/L; (**c**) temperature-range fluorescence spectrum of indicator only, 0.005 g/L; (**d**) full scale fluorescence spectrum of indicator only, 0.005 g/L, 31 °C.

To evaluate the indicator **8**, the phase transition LCST behavior of the PNIPAm copolymer was first measured in the absence of metal ion using the shift of the dansyl fluorophore’s maximum emission intensity upon increasing temperature (Figure 5). A solution containing 0.005 g/L of **8** in 0.1 M 2-(N-morpholino)ethanesulfonic acid (MES) buffer at pH 6.0 was analyzed for its emission properties. The ratio of emission intensity at 536 nm and 505 nm was monitored to observe the relationship between intensity ratio and temperature (Supplementary Materials, Figure S1). Upon increasing temperature from 25 °C to 49 °C, we observed a blue shift in maximum emission wavelength from 536 nm to 505 nm and an increase in the ratio of emission intensity (I_505 nm_/I_536 nm_) from 0.77 to 1.23. This corresponds to the expected PNIPAm thermal phase transition, whereby increasing temperature drives the polymer toward the globule state and results in a more hydrophobic environment around the dansyl fluorophore. 

The response of indicator **8** to Cu^2+^ was obtained at 0.01 mM Cu^2+^ added to 0.005 g/L of **8** in MES buffer at pH 6.0 (Figure 5). A red shift of approximately 40 nm in the maximum emission wavelength with increasing temperature from 25 °C to 49 °C is observed, indicating that Cu^2+^ produces the desired ratiometric response. Relative to the measured Cu^2+^ formation constant for the model ligand **4** of log *K*_f_ = 11.65, the amount of Cu^2+^ required for a response in these studies seems high because the corresponding dissociation constant *K*_d_ of 10^−11.65^ would indicate binding of submicromolar Cu^2+^. However, we have observed in the past that binding affinities are decreased for polymer-bound ligands, likely because a more hydrophobic environment exists which destabilizes the formation of catalytic complexes [13]. Addition of 0.01 mM Zn^2+^ to the aqueous indicator (0.005 g/L) at 25 °C yields a lesser red shift of 10 nm, consistent with our observation of a smaller K_f_ for [ZnPEPMA]^2+^ relative to [CuPEPMA]^2+^ (Figure 5).

### 2.5. Indicator Response to Aqueous Cu^2+^and Zn^2^ at Fixed Temperature^+^

**Figure 6:**
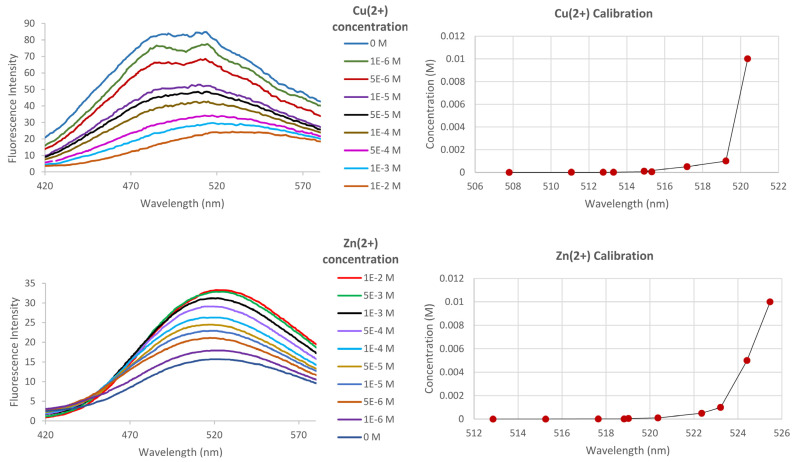
Concentration response of indicator **8** (0.1 M MES buffer, pH 6.0, 0.005 g/L) to aqueous Cu^2+^ and Zn^2+^, 45 °C. λ_ex_ = 330 nm; excitation slit width = 10; and emission width = 10.

The indicator **8** response to added Cu^2+^ and Zn^2+^ was determined via fluorescence titration at 45 °C (Figure 6). The shift of the dansyl fluorescence maxima was used to obtain calibration curves for the metal ions. The approximate limit of detection (LOD) for either metal ion is 500 µM, or approximately 31 µg/mL. 

### 2.6. Selectivity of the Metal Indicator for Cu^2+^ Relative to Ni^2+^, Fe^2+^, and Zn^2+^

**Figure 7:**
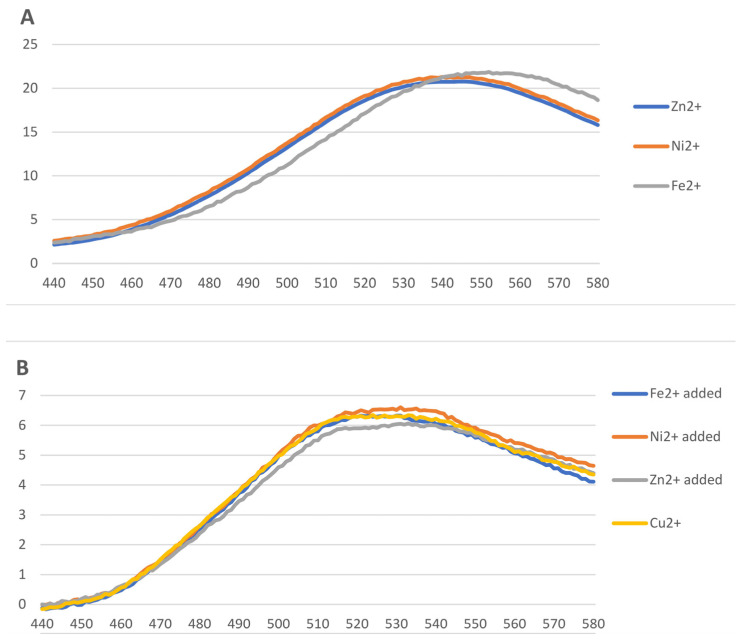
Response of indicator **8** (0.1 M MES buffer, pH 6.0, 0.005 g/L) to other metal ions in presence of Cu^2+^. (**A**) response to Zn^2+^, Ni^2+^, and Fe^2+^ alone (10 µM each). (**B**) response of competing metal ions against Cu^2+^, showing response to Cu^2+^ and response from addition of Fe^2+^, Ni^2+^, or Zn^2+^, each 10 µM. λ_ex_ = 330 nm; excitation slit width = 10; and emission width = 10.

Indicator selectivity for Cu^2+^ was studied via addition of a competing metal ion to the Cu^2+^-indicator bound form. First, the ability of the indicator to respond to Ni^2+^ and Fe^2+^ was checked (Figure 7A), indicating that the fluorescence maximum of Ni^2+^ was similar to Zn^2+^ at ca. 540 nm and the maximum of Fe^2+^ at 550 nm. Next, a solution of the Cu^2+^-complexed indicator was treated with an equal concentration of one of the ions Fe^2+^, Ni^2+^, or Zn^2+^. No change in the fluorescence response of the indicator was observed, consistent with the inability of these ions to compete with or disturb the response to copper(II). 

### 2.7. Reversibility of the Metal Indicator

**Figure 8:**
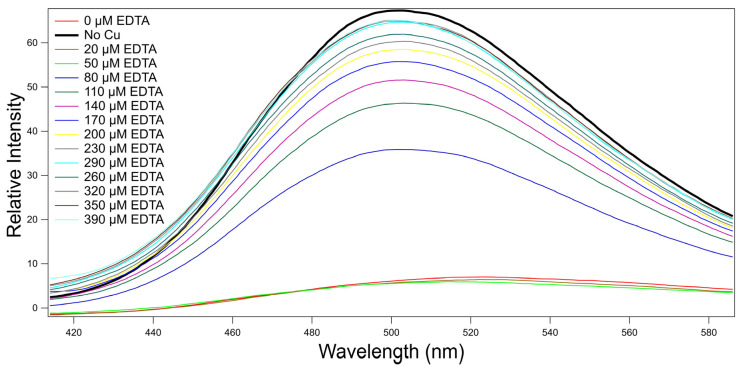
Fluorescence spectra of the polymer sample with 10 μM Cu^2+^ titrated with different concentration of EDTA. λ_ex_ = 330 nm; excitation slit width = 10; and emission width = 10. Concentration of the sample: 0.005 g/L in 0.1M MOPS buffer pH 7.0, 35 °C.

To assess the suitability of this indicator for dynamic measurements of metal ion concentration, reversibility of the response was studied. Ethylenediaminetetraacetic acid (EDTA), a Cu^2+^ chelating agent, was added to 0.005 g/L **8** containing 10 μM Cu^2+^. Below the LCST, one equivalent of EDTA was able to essentially quantitatively remove the Cu^2+^ from **8** (Supplementary Materials, Figure S1) within a time frame of 5 min. However, at and above the LCST, an excess concentration of about 15-fold EDTA was needed in order to regain comparable fluorescence emission intensities, for which a time of about 25 min was required (Figure 8). This was expected because the globular polymer form can present a great kinetic barrier to Cu^2+^ removal relative to the coiled form. This demonstrates the reversibility of Cu^2+^ binding and suggests that the system may be used to detect slow changes in Cu^2+^ concentrations at 35 °C and more rapid changes at 25 °C. Additionally, this supports the hypothesis that **8** may be regenerated with EDTA treatment and used for subsequent testing. 

## 3. Discussion and Conclusions

This work has demonstrated a novel ratiometric indicator of Cu^2+^ and Zn^2+^ based on a high-affinity ligand that modulates polymer conformation upon metal binding, resulting in a shift in maximum fluorescence. Due to the quenching nature of the Cu^2+^ ion, many studies have sought to create turn-on sensors based on structural changes in the sensor. Thus, for example, a BODIPY-adamantyl fluorophore is quenched via self-assembly with bovine serum albumin (BSA) into nanoparticles, which, in turn, are decomplexed by Cu^2+^, resulting in a turn-on response to copper(II) [23]. This unique system is of interest for biological environments which do not denature BSA. Another useful strategy is an activity-based one, in which Cu^2+^ causes or allows a chemical transformation that may lead to a fluorescence enhancement. Fluorescent spirobifluorenes form complexes with borderline-to-soft bases, including Cu^2+^, Hg^+^, and Pb^2+^, with resultant quenching that can be reversed by competitive complexation with cyanide, which allows nonselective Cu^2+^ sensing but requires (or also detects) the cyanide ion [24]. Unlike many irreversible copper-mediated reactions, the complexation in this system is reversible, allowing for dynamic concentration sensing. 

Our PEPMA-C_3_-ligand-based indicator **8** has a measured Cu^2+^ affinity of ca. log 11, which is compatible with amounts of bioavailable Cu^2+^ in natural waters and the expected preference for Cu^2+^ over Zn^2+^. The present work studied zinc detection only as a control metal ion for the sensor, but many turn-on zinc sensors exist because Zn^2+^ does not quench fluorescence [25,26,27]. The present sensor is reversible, and therefore can be viable for use in various aqueous Cu^2+^ systems where concentrations can fluctuate. The suitability for use in natural waters will require possible adjustments to this system; for example, an increase in the amount of chelator in the PNIPAm framework may yield greater sensitivity and a more rapid response. The present studies give insight towards the design of an indicator with potential application in monitoring Cu^2+^ concentration in diverse systems, and with the potential to regenerate the system through treatment with a small molecule chelator.

## 4. Materials and Methods

### 4.1. General 

All materials listed below were of research grade or a spectro grade in the highest purity available and were generally used without purification except 2-pyridinecarboxaldehyde, which was distilled, and acrylonitrile, which was passed through a plug of basic alumina. ^1^H and proton-decoupled ^13^C NMR were obtained using a Varian Mercury 400 MHz NMR instrument, and chemical shifts are reported in ppm relative to the deuterated solvent used. Elemental analysis was performed by Atlantic Microlabs (Atlanta, GA, USA). Mass spectral data were obtained on a Bruker AmaZon SL ion trap LC/MS (Northwestern University). X-ray crystallography was performed on a Bruker APEX-II CCD Diffractometer (UC San Diego). Potentiometric titrations were performed using a 785 DMP Titrino equipped with an Accumet double injection pH electrode and thermally regulated titration vessel. Formation-constant data were evaluated using the Hyperquad2008 program suite [28]. The fluorescence spectra were recorded using a Cary Eclipse fluorescence spectrophotometer with 3 cm^3^ quartz cuvette (1 cm pathlength). 

Caution! Perchlorates of metal complex cations have been known to explode. No explosions occurred during this work. 

### 4.2. Synthesis of Compounds

#### 4.2.1. N-(2-pyridinylethyl)-2-pyridinemethanamine (PEPMA) **(1)**

2-(2-ethylamino)pyridine (2.10 g, 17.2 mmol) and 2-pyridinecarboxaldehyde (2.018 g, 18.87 mmol) were combined in MeOH (30 mL) and stirred at room temperature under nitrogen atmosphere for 2 h. Then, sodium borohydride (0.705 g, 18.6 mmol) was added slowly and the mixture was stirred at room temperature for 24 h. After 24 h, the yellow solution was concentrated under reduced pressure and the resulting yellow residue was dissolved in water (ca. 20 mL). The obtained solution was acidified to pH ca. 4 via dropwise addition of 6 M HCl. The aqueous layer was extracted with CHCl_3_ (2 × 20 mL). Then, the aqueous layer was basified to pH ca. 11 via dropwise addition of 2 M NaOH. The basic solution was extracted with CHCl_3_ (3 × 15 mL). The extracted organic layer was dried over magnesium sulfate, vacuum filtered, concentrated via rotary evaporator, and dried to yield the product as a brown oil (3.223 g, 15.11 mmol, 88%). ^1^H NMR (400 MHz, CDCl_3_) δ 8.54 (d, *J* = 4.9 Hz, 2H), 7.60 (d, *J* = 7.78 Hz, 2H), 7.34 (m, 1H), 7.30 (m, 3H), 3.95 (s, 2H), 3.06 (m, 4H), 2.14 (s, N,H 1H).^13^C NMR (101 MHz, CDCl_3_): 160.40, 160.10, 149.50, 149.43, 136.54, 136.46, 123.43, 123.31, 121.99, 121.37, 55.24, 49.14, 38.72; Exact mass calculated for C_13_H_15_N_3_ [M-H]^−^, 213.13. Found, 213.98.

#### 4.2.2. 3-((2-(pyridin-2-yl)ethyl)(pyridin-2-ylmethyl)amino)propionitrile **(2)**

Acrylonitrile (0.687 g, 12.8 mmol) was filtered through a plug of basic alumina and added to a 100 mL round bottom flask equipped with a Teflon-coated stir bar. Ligand **1** (1.3702 g, 6.14 mmol) and MeOH (30 mL) were added to the flask, respectively. The reaction mixture was refluxed under N_2_ atmosphere for 72 h. After cooling, the resulting mixture was concentrated via rotary evaporator and vacuum dried to yield the product as a brown oil (1.524 g, 5.726 mmol, 93%). ^1^H NMR (400 MHz, CDCl_3_): 8.51 (2H, m), 7.61 (2H, m), 7.39 (1H, m), 7.15 (3H, m), 3.87 (2H, s), 3.02 (4H, m), 2.89 (2H, t, *J* = 6.8 Hz), 2.45 (2H, t, *J* = 6.9 Hz). ^13^C NMR (101 MHz, CDCl_3_): 160.05, 159.25, 149.76, 149.45, 149.12, 136.82, 136.55, 123.72, 123.11, 122.40, 119.04, 64.55, 60.30, 54.11, 49.81, 36.25, and 16.67; Exact mass calculated for C_16_H_18_N_4_ [M-H]^−^, 266.15. Found, 266.07.

#### 4.2.3. *N*-(2-(pyridin-2-yl)ethyl)-*N*-(pyridin-2-ylmethyl)propane-1,3-diamine **(3)**

To a 50% suspension of active Raney-Ni (8.1 g) in water (ca. 190 mL), we added a solution of **2** (13.84 g, 52 mmol) in MeOH (225 mL). The resulting mixture was vigorously stirred and NaBH_4_ (4.04 g, 106 mmol) in 8 M of NaOH (57 mL) was added at such a rate as to maintain temperature at 60 °C. After the addition, the reaction was stirred overnight at room temperature. Raney-Ni catalyst was removed via filtration through Celite and the solvent was removed under reduced pressure. A portion of 8 M NaOH (57 mL) was added subsequently to the residue, which was then extracted with CH_2_Cl_2_ (5 × 50 mL). The organic layers were combined and dried over anhydrous Na_2_SO_4_. After filtration, the organic layer was concentrated via rotary evaporator and vacuum dried to yield the product as brown oil (7.500 g, 27.73 mmol, 53.35%). ^1^H NMR (400 MHz, CDCl_3_): 8.51 (2H, d, *J* = 5.0 Hz), 7.57 (2H, d, *J* = 7.6 Hz), 7.33 (1H, m), 7.11 (3H, t, *J* = 7.4 Hz), 3.80 (2H, s), 2.94 (4H, m), 2.61 (4H, m), 1.61 (2H, m). ^13^C NMR (101 MHz, CDCl_3_): 160.82, 160,52, 149.34, 149.02, 136.44, 136.28, 123.46, 122.89, 121.913, 121.21, 60.71, 54.49, 51.85, 40.35, 36.07, 31.32; Exact mass calculated for C_16_H_22_N_4_ [M − H]^−^, 270.18. Found, 271.09.

#### 4.2.4. *N*-(3-((2-(pyridin-2-yl)ethyl)(pyridin-2-ylmethyl)aminopropylisobutyramide **(4)**

Triethylamine (8.0 mL, 56 mmol) and **3** (4.0 g, 12 mmol) were dissolved in CH_2_Cl_2_ (80 mL). The resulting mixture was stirred in at 0 °C for 10 min. Isobutyryl chloride (1.36 mL, 13.0 mmol) was then added slowly and the reaction was stirred at room temperature. After 24 h, the reaction mixture was dissolved in 0.1 M HCl (150 mL) and extracted with CH_2_Cl_2_ (3 × 50 mL). The organic layers were combined, dried with anhydrous Na_2_SO_4_, filtered, and concentrated via rotary evaporator to yield crude product, which was then purified with alumina column chromatography (EtOAc:hexanes 9:1) to afford the product as a yellow oil (1.787 g, 30%). TLC (alumina), R*_f_* = 0.30. ^1^H NMR (400 MHz, CDCl_3_): 8.48 (2H, m), 7.66 (1H, t, *J* = 5.7 Hz), 7.56 (1H, t, *J* = 7.7 Hz), 7.47 (1H, m), 7.14 (2H, m), 7.05 (1H, d, *J* = 7.8 Hz), 6.97 (1H, d, *J* = 7.8 Hz) 3.70 (2H, s,), 3.25 (2H, t, *J* = 5.9 Hz), 2.95 (2H, t, *J* = 6.6 Hz), 2.85 (2H, t, *J* = 6.6 Hz), 2.66 (2H, t, *J* = 6.2 Hz), 2.43 (1H, m), 1.70 (2H, t, *J* = 6.2 Hz), 1.15 (6H, d, *J* = 6.9 Hz). ^13^C NMR (101 MHz, CDCl_3_): 177.20, 160.63, 159.65, 148.89, 148.67, 136.38, 136.26, 123.33, 122.76, 121.88, 121.18, 59.90, 54.65, 51.24, 37.40, 35.67, 35.36, 26.11, 19.79; Exact mass calculated for C_20_H_28_N_4_O [M − H]^−^, 340.23. Found, 340.19.

#### 4.2.5. *N*-(3-((2-(pyridin-2-yl)ethyl)(pyridin-2-ylmethyl)aminopropylacrylamide (PEPMA-C3-acrylamide) **(5)**

Triethylamine (7.0 mL, 51 mmol) and **3** (3.00 g, 11 mmol) were dissolved in CH_2_Cl_2_ (75 mL) and stirred at 0 °C for 10 min before adding acryloyl chloride (1.07 g, 11.88 mmol). The resulting mixture was stirred at room temperature for 24 h. The solvent was removed under reduced pressure and the resulting crude mixture was washed with 0.1 M HCl (150 mL). The aqueous layer was extracted with CH_2_Cl_2_ (3 × 50 mL), and the organic layers were dried with anhydrous Na_2_SO_4_. After filtration, the organic layer was concentrated via rotary evaporator to yield crude product, which was then purified by alumina column chromatography (EtOAc, 100%) to afford the product as a yellow oil (1.32 g, 37%). TLC (alumina) R*_f_* = 0.38. ^1^H NMR (400 MHz, CDCl_3_): 8.47 (2H, d, *J* = 4.9 Hz), 7.55 (1H, t, *J* = 7.7 Hz), 7.43 (1H, t, *J* = 7.7 Hz), 7.12 (2H, m), 7.03 (1H, d, *J* = 7.8 Hz), 6.88 (1H, d, *J* = 7.8 Hz), 6.29 (2H, m), 5.57 (1H, dd, *J* = 8.7 Hz), 3.65 (2H, s), 3.35 (2H, t, *J* = 5.9), 2.93 (2H, m), 2.82 (2H, t, *J* = 6.7), 2.69 (2H, t, *J* = 6.0 Hz), 1.76 (2H, t, *J* = 6.0 Hz). ^13^C NMR (101 MHz, CDCl_3_): 165.94, 160.85, 159.70, 149.14, 148.74, 136.45, 136.45, 131.82, 125.32, 123.57, 122.91, 122.09, 121.42, 59.97, 55.04, 51.58, 37.84, 35.91, 25.88; Exact mass calculated for C_19_H_24_N_4_O [M − H]^−^, 324.20. Found, 324.16.

#### 4.2.6. *N*-(2-aminoethyl)-5-(dimethylamino)naphthalene-1-sulfonamide **(6)**

A solution of ethylenediamine (5.560 mL, 83.00 mmol) and dry THF (20 mL) was cooled at 0 °C. After 10 min, dansyl chloride (0.500 g, 1.85 mmol) in dry THF (5 mL) was added dropwise over a period of 1.5 h. After stirring at room temperature for 2 h, 1.0 M NaOH (5 mL) was added. THF was removed on the rotary evaporator and the aqueous solution was extracted with CH_2_Cl_2_ (3 × 20 mL). The combined organic extracts were dried over anhydrous Na_2_SO_4_ and filtered. The solvent was removed under reduced pressure and the resulting crude material was recrystallized from a 1:1 benzene: hexanes mixture to afford a light-yellow solid (0.187 g, 34%). ^1^H NMR (400 MHz, CDCl_3_) δ 8.54 (dt, *J* = 8.5, 1.1 Hz, 1H), 8.34–8.22 (m, 2H), 7.55 (m, 2H), 7.19 (dd, *J* = 7.6, 0.9 Hz, 1H), 2.89 (m, 8H), 2.70 (m, 2H). ^13^C NMR (101 MHz, CDCl_3_) δ 152.25, 134.86, 130.65, 130.11, 129.92, 129.83, 128.62, 123.42, 118.90, 115.42, 45.73, 45.64, 41.00.

#### 4.2.7. *N*-(2-(5-(dimethylamino)naphthalene-1-sulfonamido)ethyl)acrylamide **(7)**

Ligand **6** (0.1868 g, 0.605 mmol) and triethylamine (0.0677 g, 0.670 mmol) were dissolved in dry CH_2_Cl_2_ (50 mL). The resulting mixture was cooled at 0 °C, and acryloyl chloride (0.0606 g, 0.669 mmol) was added dropwise. After stirring for 5 h at room temperature, the precipitate was removed by filtration and the filtrate was concentrated under reduced pressure to yield crude product, which was then purified via silica gel column chromatography (EtOAc, 100%) to afford a yellow solid (0.1425 g, 68%). ^1^H NMR (400 MHz, CDCl_3_) δ 8.54 (d, *J* = 8.9 Hz, 1H), 8.25 (d, *J* = 8.9 Hz, 2H), 7.60–7.47 (m, 2H), 7.17 (d, *J* = 7.6 Hz, 1H), 6.34 (t, NH, *J* = 5.8 Hz, 1H), 6.16 (dt, *J* = 16.9, 0.9 Hz, 1H), 5.97–5.85 (m, 2H), 5.58–5.50 (m, 1H), 3.38 (dt, *J* = 5.7 Hz, 2H), 3.07 (dt, *J* = 5.8 Hz, 2H), 2.88 (d, *J* = 0.5 Hz, 6H). ^13^C NMR (101 MHz, CDCl_3_) δ 166.53, 152.31, 134.44, 130.94, 130.61, 130.15, 129.89, 129.68, 128.85, 126.95, 123.42, 118.80, 115.52, 45.64, 43.28, 39.58.

#### 4.2.8. [Cu(**4**)](ClO_4_)_2_•0.5 H_2_O

Cu(ClO_4_)_2_•6H_2_O (0.025 g, 0.067 mmol) in MeOH (2 mL) was added to ligand **4** (0.023 g, 0.068 mmol) in MeOH (2 mL), causing a color change from pale blue to deep blue. Vapor diffusion of Et_2_O into this solution (24 h) afforded blue crystals. These were isolated via decantation of solvent and air-dried at ambient temperature, yield 0.029 g (70%). Anal. Calcd (%) for C_20_H_29_Cl_2_CuN_4_O_9_._5_: C, 39.25; H, 4.74; N, 9.16. Found: C, 39.01; H, 4.52; N, 9.01. UV (MeOH) 698 nm (ε = 110.5) MS *m/z* 503.32 (M-ClO_4_-0.5 H_2_O).

### 4.3. Polymer Synthesis

General procedure for polymerization: NIPAM (0.9261 g, 8.184 mmol), PEPMA-C3-acrylamide **5** (0.1427 g, 0.44 mmol), dansyl acrylamide **7** (0.06115 g, 0.176 mmol), 2-(dodecylthiocarbonothioylthio)-2-methylpropionic acid (3.21 mg, 8.8 μmol), and azobisisobutyronitrile (AIBN) (0.1445 mg, 0.88 μmol) were dissolved in DMF (5 mL). The resulting solution was purged with N_2_ for 30 min at room temperature and brought to 70 °C for 24 h. The resulting polymer was dialyzed in water for 3 days. Water was removed and the polymer was dried on the vacuum before characterization with ^1^H NMR and DOSY [19] NMR. 

### 4.4. Structural Studies

Single crystals of C_40_H_58_Cl_4_Cu_2_N_8_O_19_ were obtained by vapor-phase diffusion of Et_2_O into a MeOH solution of the complex. A suitable crystal was selected and mounted on a Bruker APEX-II CCD diffractometer. The crystal was kept at 100.0 K during data collection. Using Olex2 [29], the structure was solved with the ShelXS [30] structure solution program using direct methods and refined with the ShelXL [31] refinement package using least squares minimization.

## Data Availability

Data is in supporting information.

## Supplementary Materials

The following supporting information can be downloaded at https://www.mdpi.com/article/10.3390/molecules28207097/s1: ^1^H NMR, ^13^C NMR, and DOSY spectral plots; fluorescence spectra, and crystal structure data. The structure data are also deposited with The Cambridge Crystallographic Data Centre: CCDC 2221208 contains the supplementary crystallographic data for this paper. The data can be obtained free of charge from The Cambridge Crystallographic Data Centre via www.ccdc.cam.ac.uk/structures, accessed 14 October 2023.
