# Supplementary material for: Development of a Ratiometric Fluorescent Cu(II) Indicator Based on Poly(N-isopropylacrylamide) Thermal Phase Transition and an Aminopyridyl Cu(II) Ligand
Source: Molecules. 2023 Oct 15;28(20):7097. doi: 10.3390/molecules28207097 (PMC10609634; doi:10.3390/molecules28207097)
Supplement: Supplementary file 1 [file molecules-28-07097-s001.zip › molecules-2150303-supplementary.pdf]

SUPPORTING INFORMATION

Development of a Ratiometric Fluorescent Cu(II) Indicator Based on poly(*N*-isopropylacrylamide) Thermal Phase Transition and an Aminopyridyl Cu(II) Ligand

Lea Nyiranshuti [1,†], Emily R. Andrews [1,‡], Leonid I. Povolotskiy [1,‡,§], Frances M. Gomez [1], Nathan R. Bartlett [1], Arun Timothy Royappa [2], Arnold L. Rheingold [3], William Rudolf Seitz [1] and Roy P. Planalp [1,*]

I. ^{1}H NMR, ^{13}C NMR and DOSY spectral plots – Page 2

II. Fluorescence spectra – Page 10

III. Job's plot of Cu^{2+}-PEPMA system - Page 11

IV. Crystal structure data – Page 12

I. ^{1}H NMR, ^{13}C NMR and DOSY spectral plots

Compound **1** ^{1}H-NMR

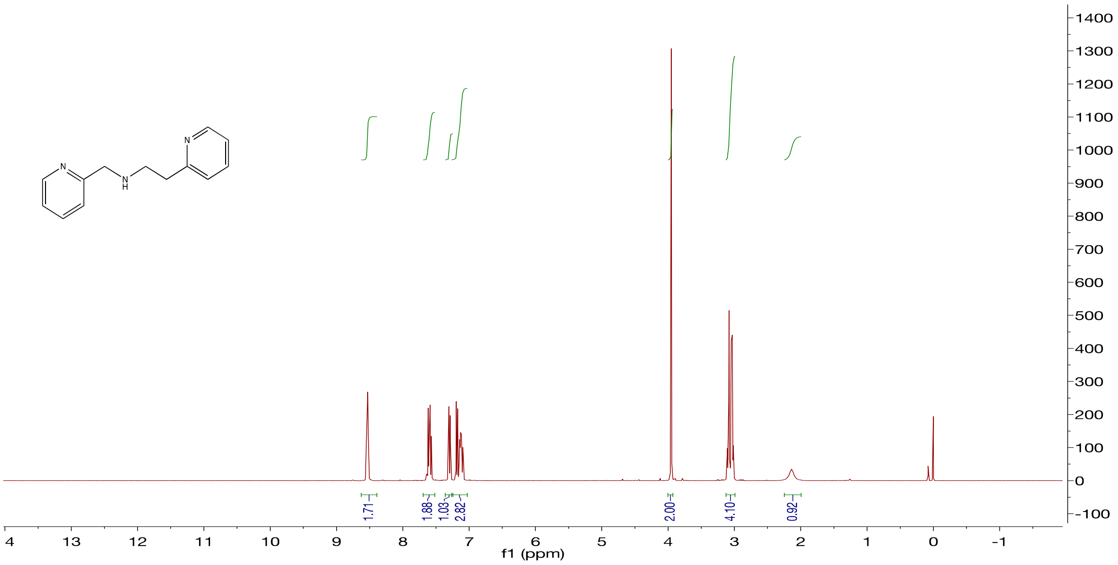

Compound **1** ^{13}C-NMR

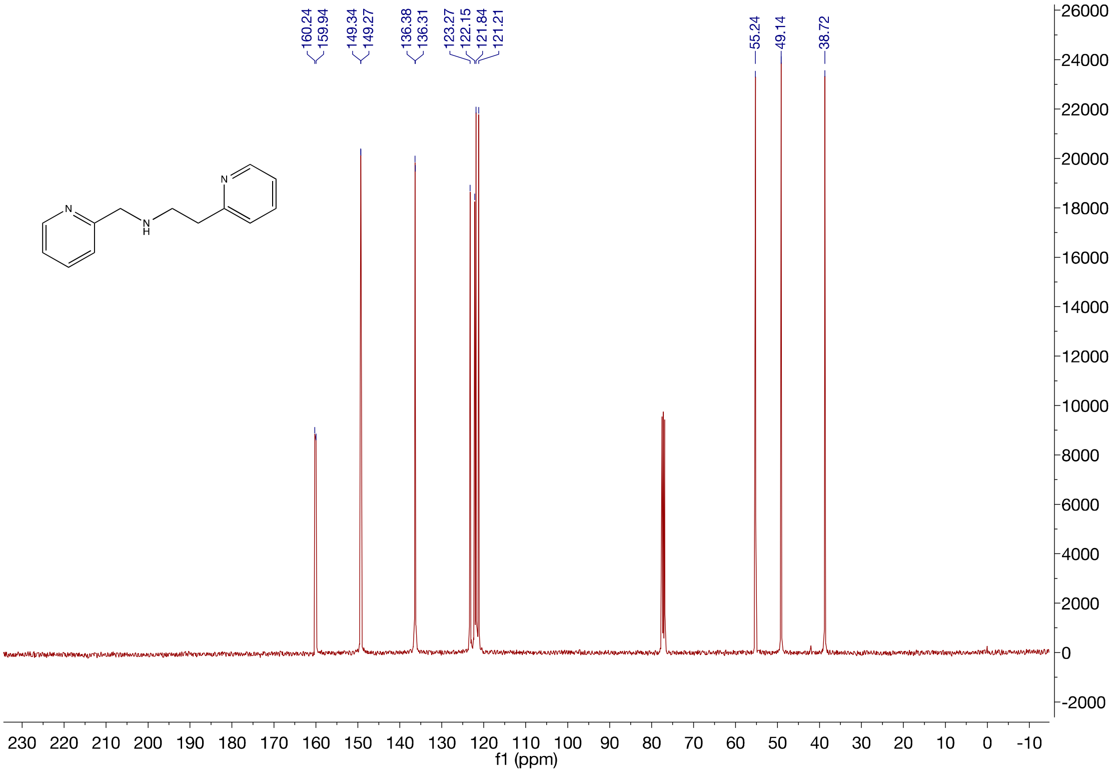

Compound **2** ^{1}H-NMR

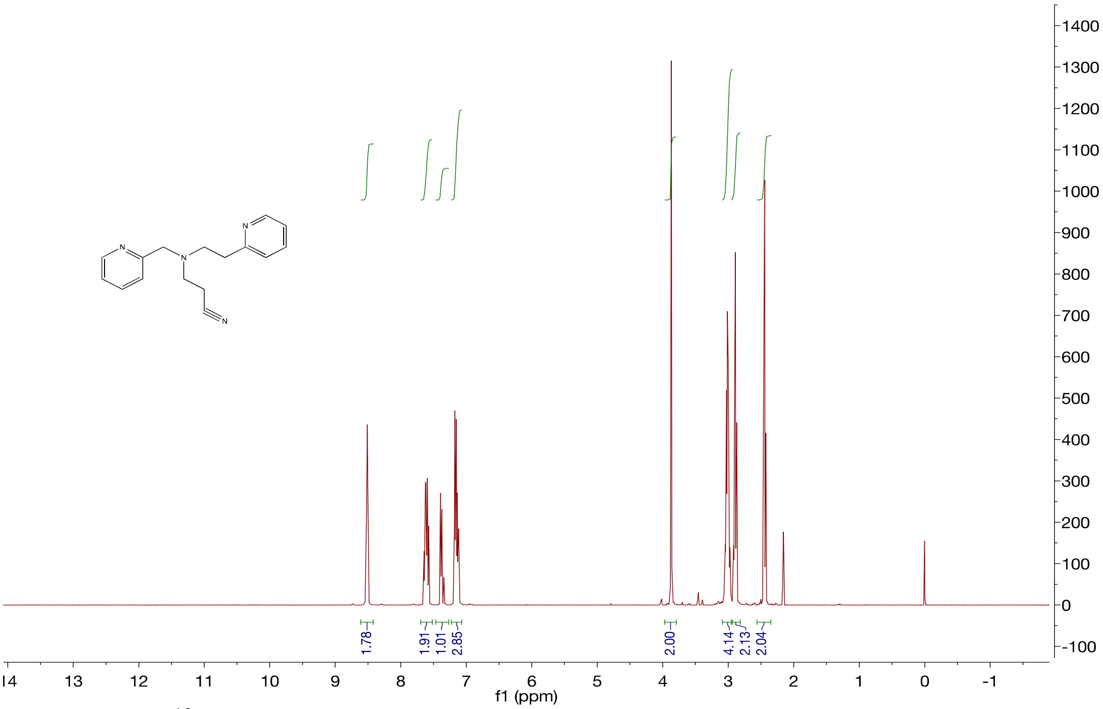

Compound **2** ^{13}C-NMR

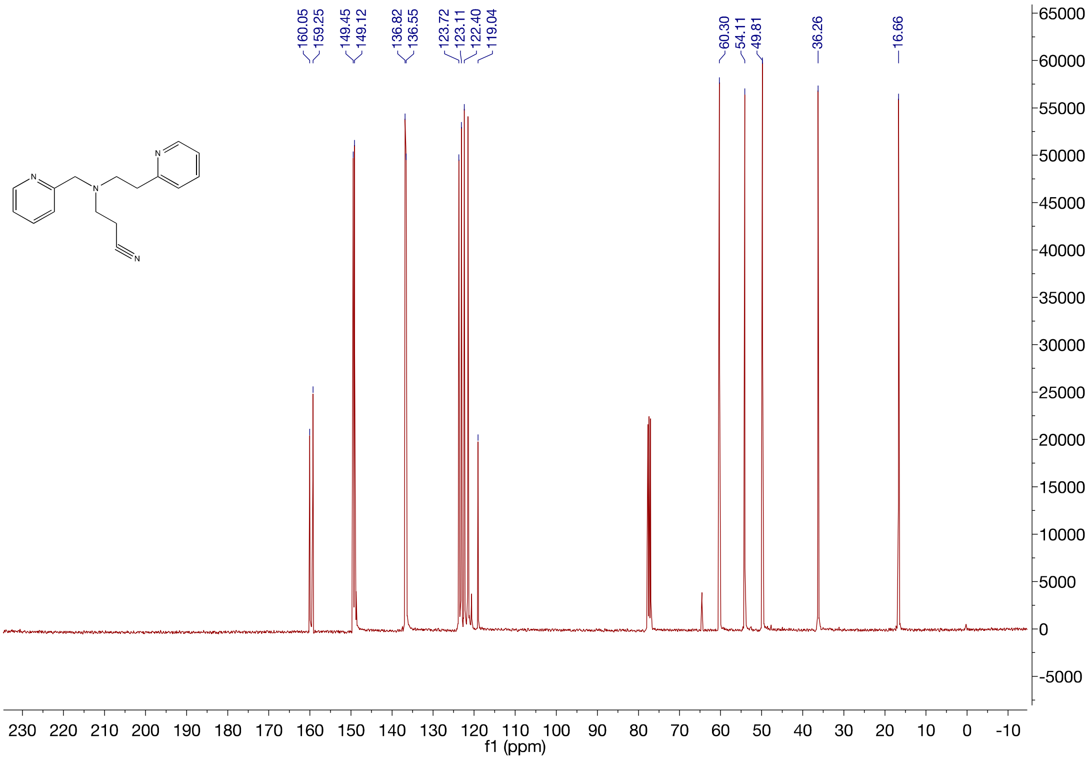

Compound **3** ^{1}H-NMR

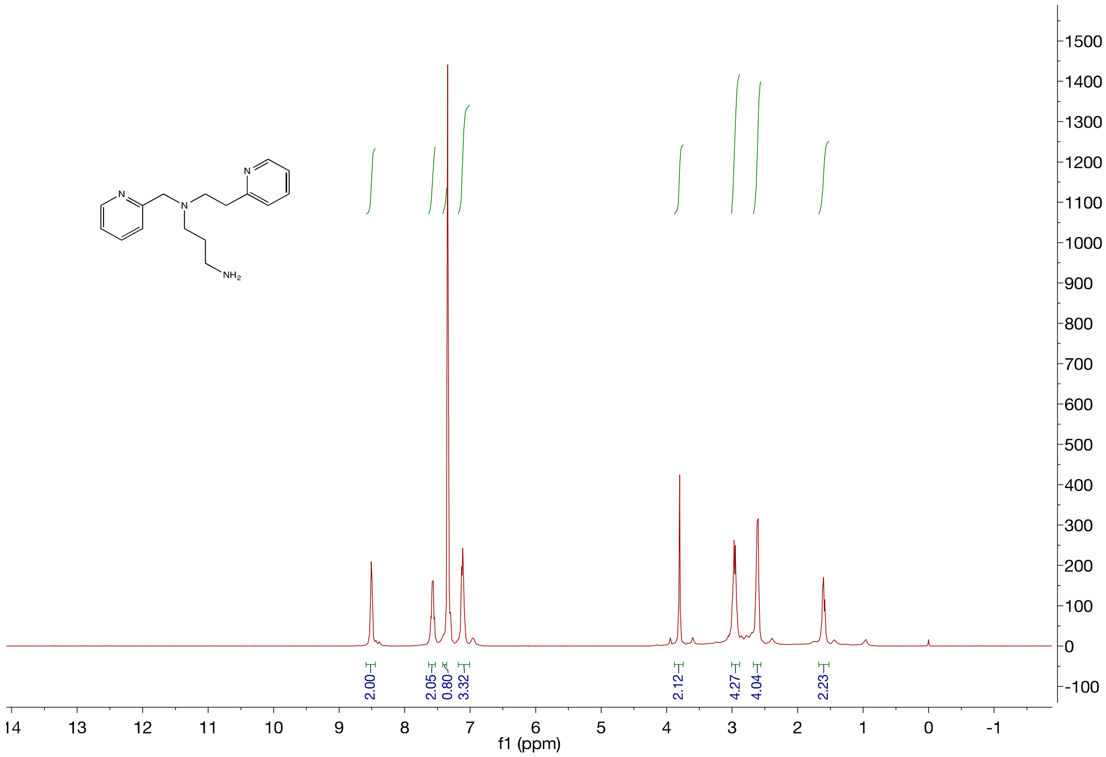

Compound **3** ^{13}C-NMR

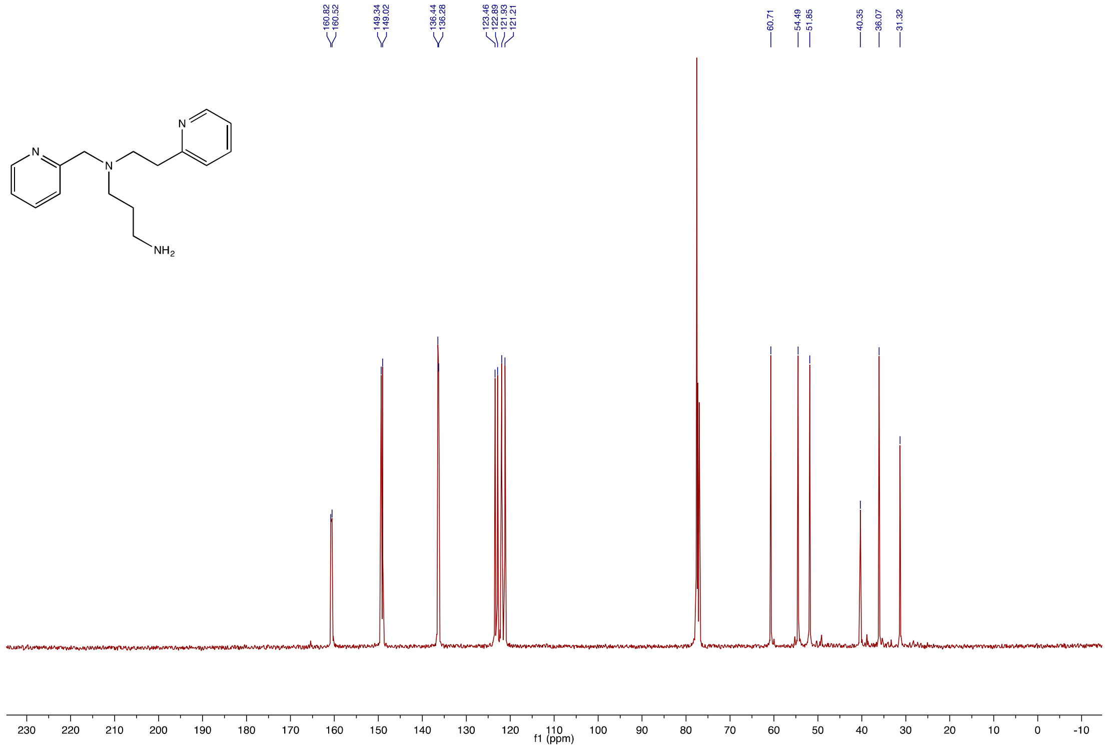

Compound **4** ^{1}H-NMR

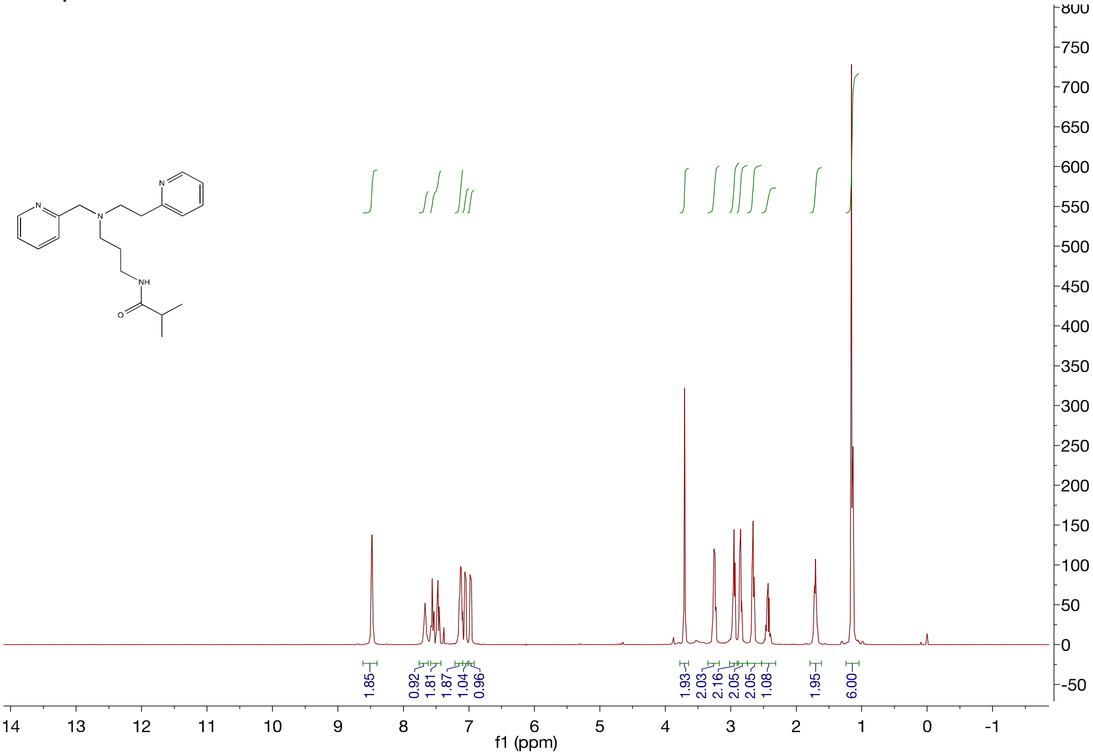

Compound **4** ^{13}C-NMR

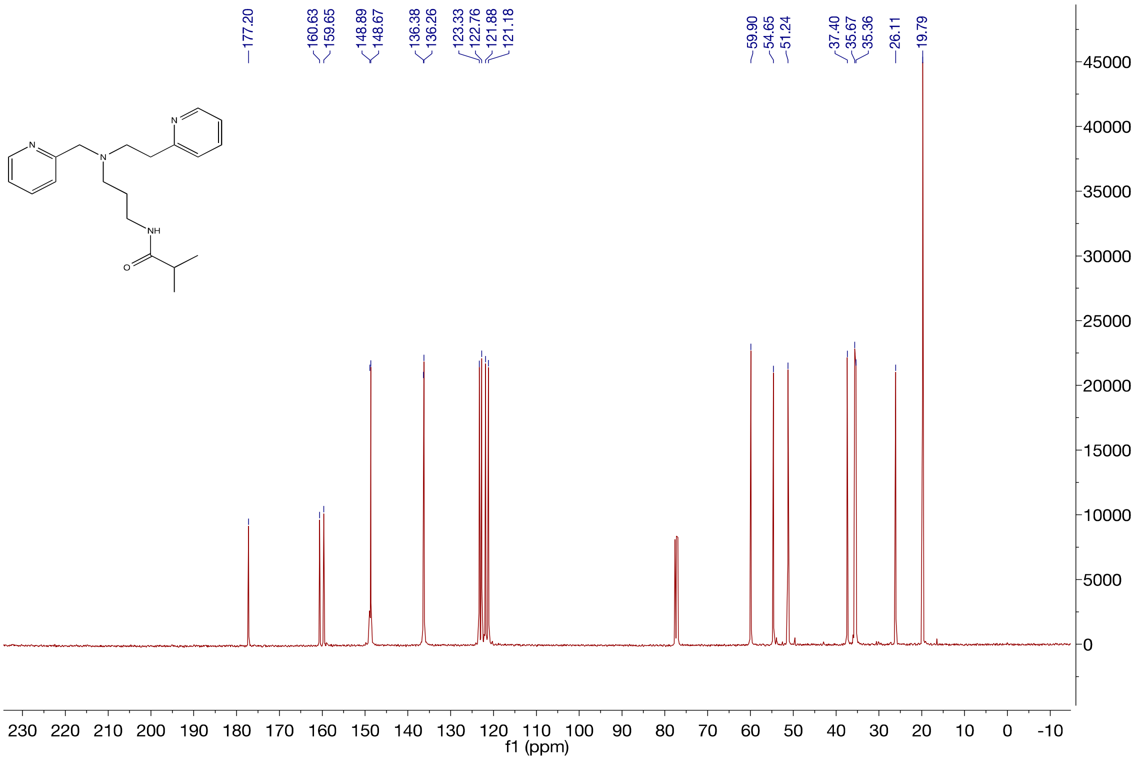

Compound **5** ^{1}H-NMR

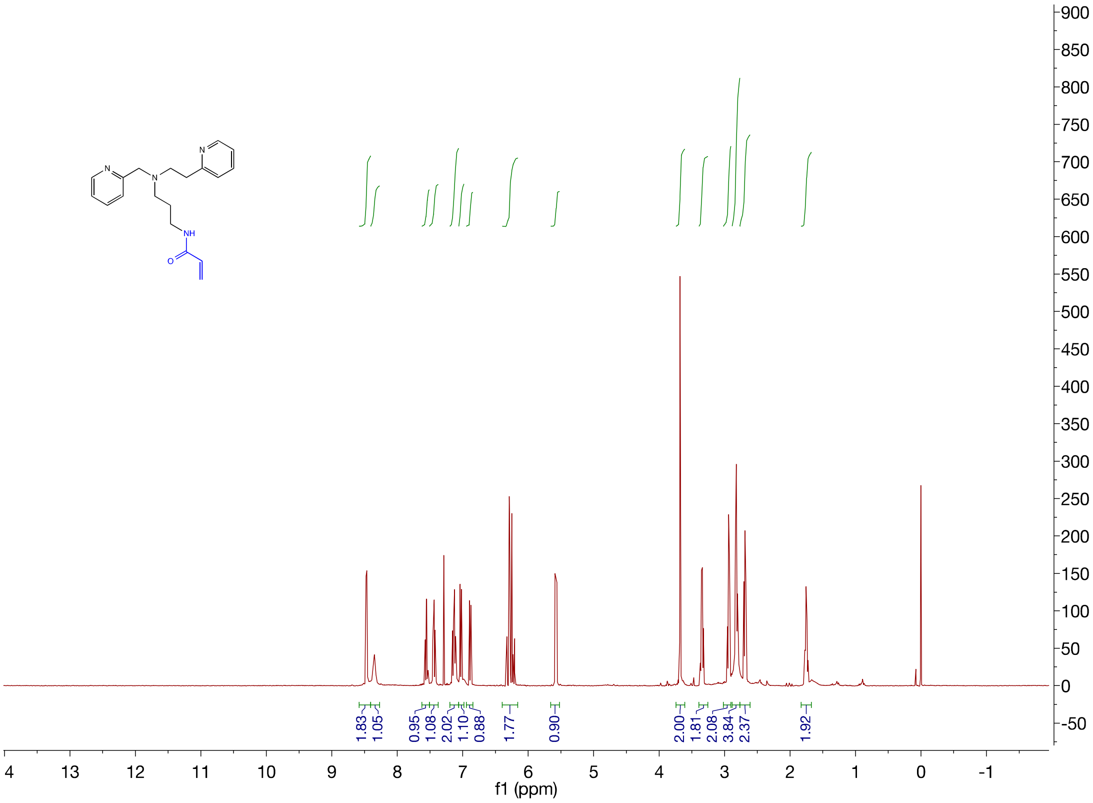

Compound **5** ^{13}C-NMR

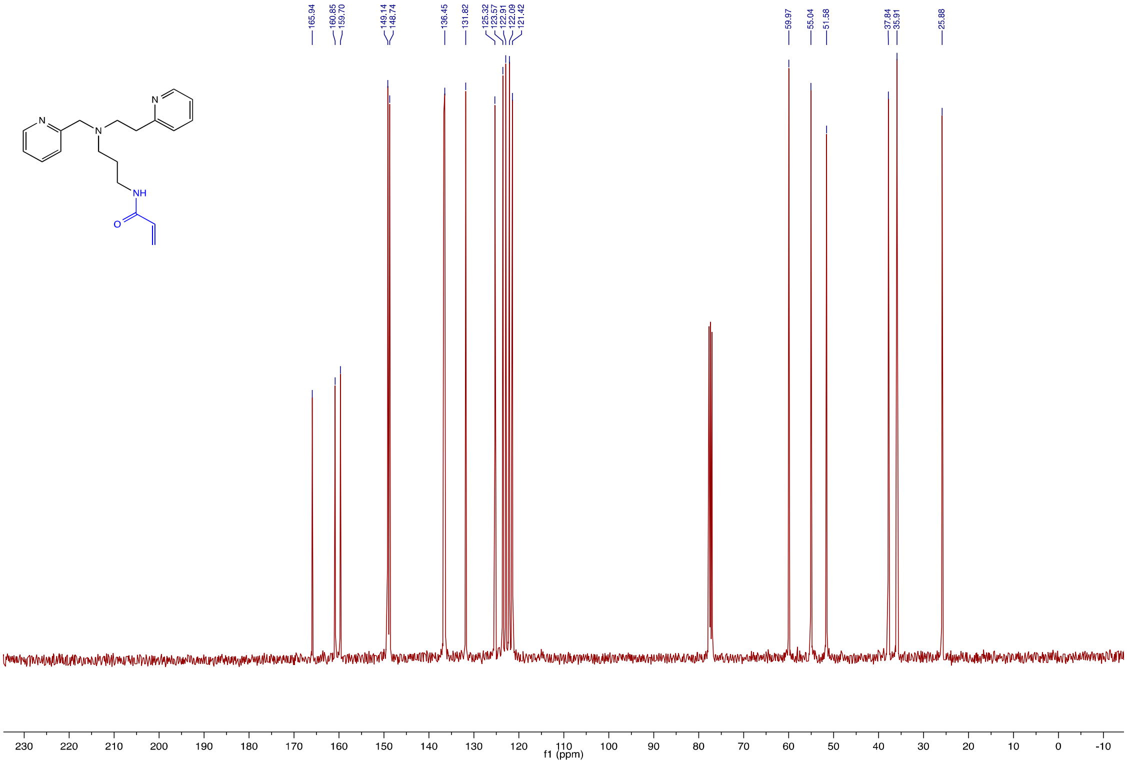

Compound **6** ^{1}H-NMR

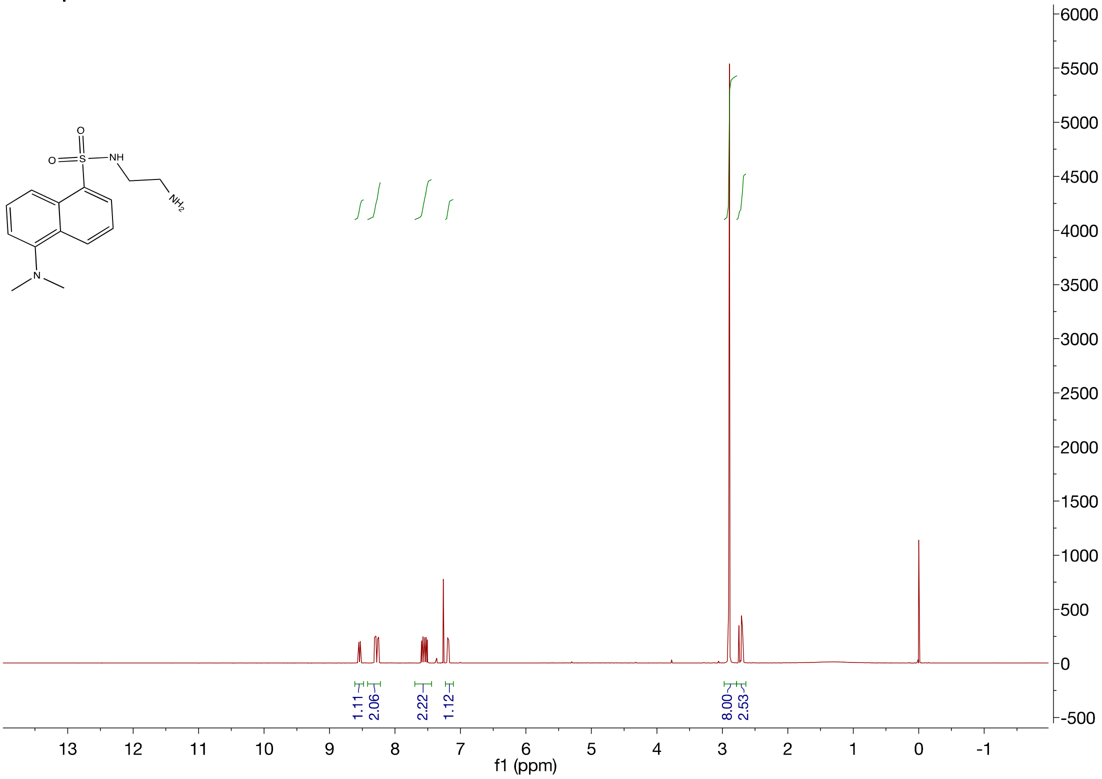

Compound **6** ^{13}C-NMR

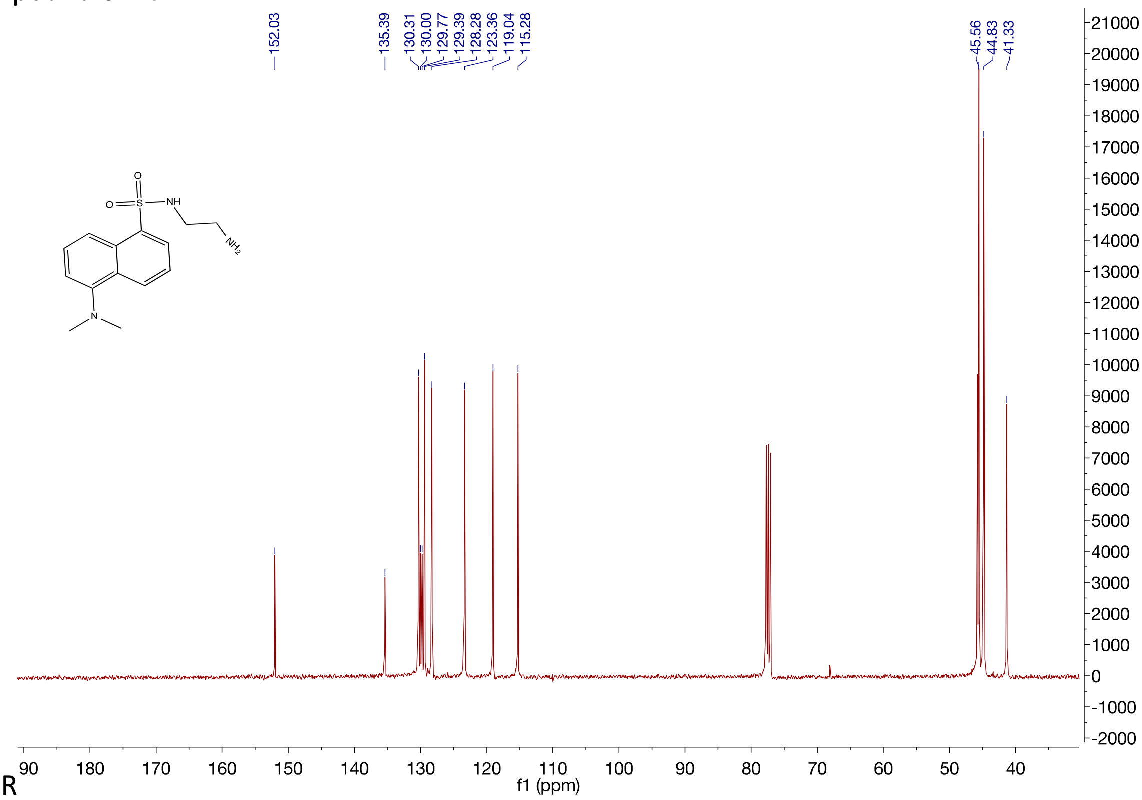

Compound **7** ^{1}H-NMR

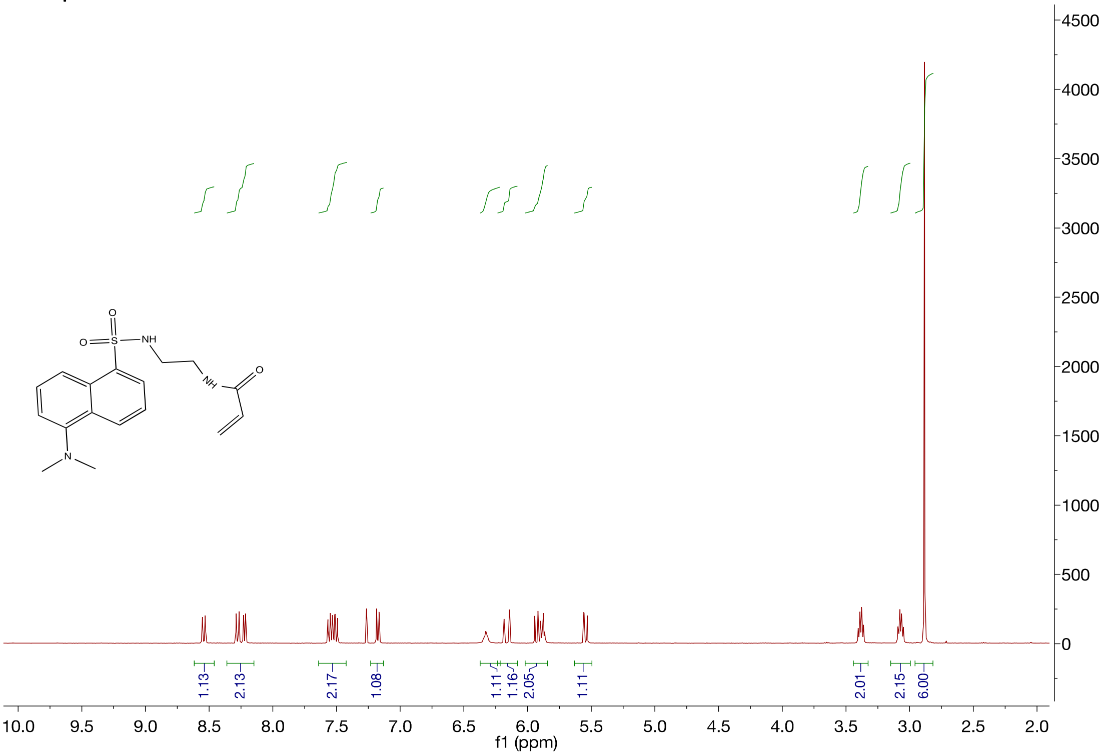

Compound 7 ^{13}C-NMR

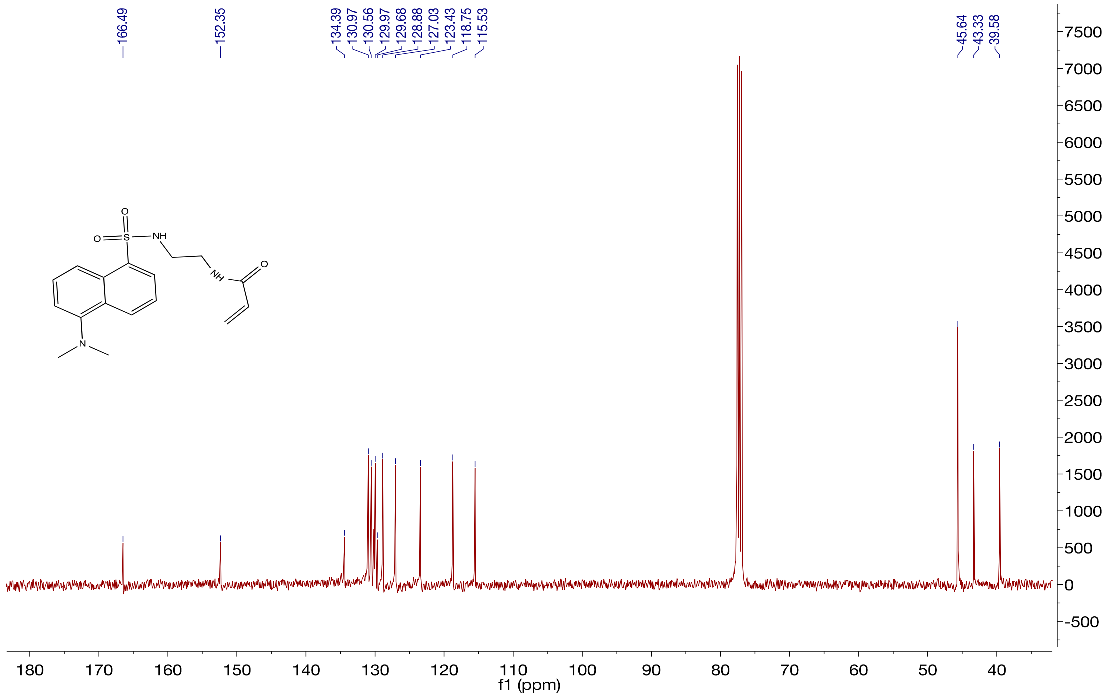

Compound **8** ^{1}H-NMR

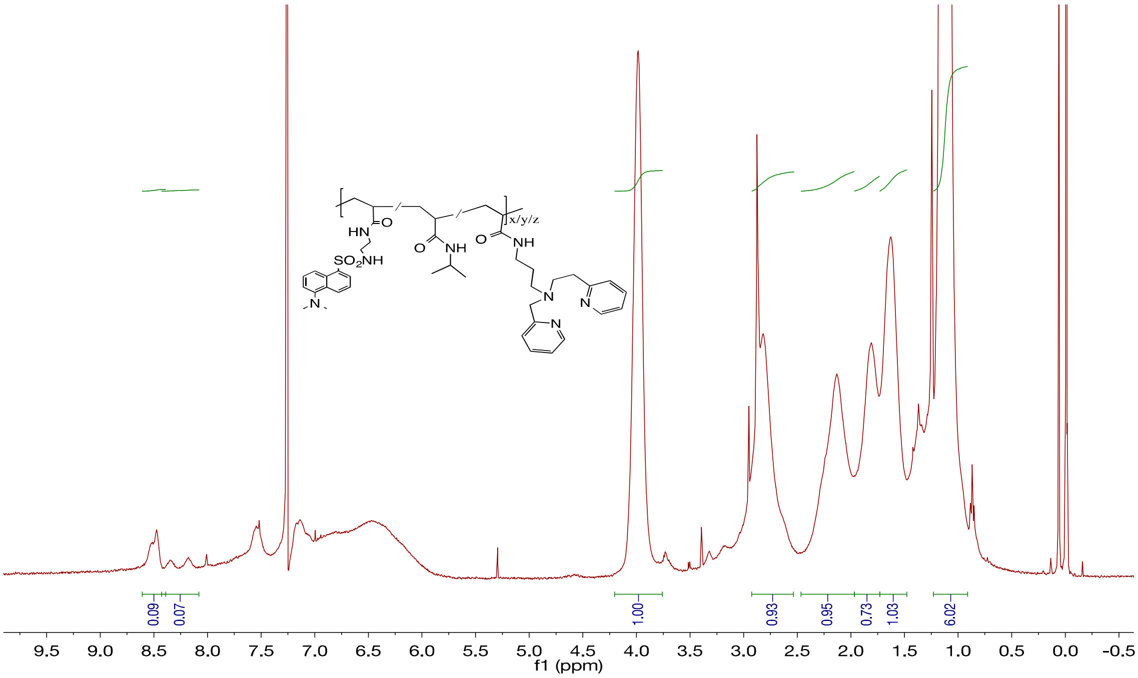

Compound **8** DOSY

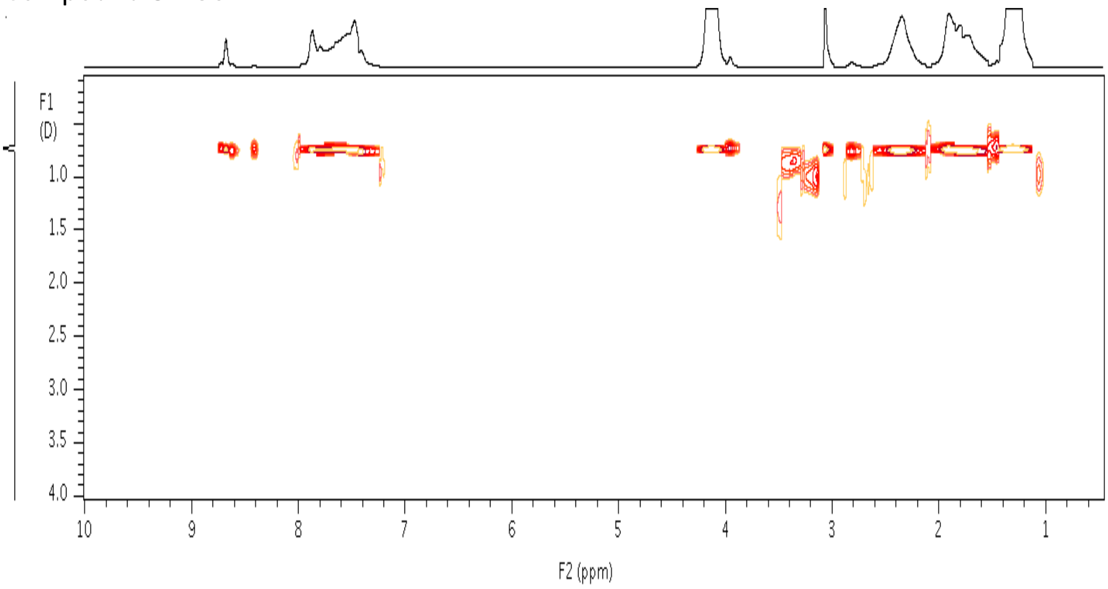

The mol fraction of **5** was calculated from the ratio of the area of the peak at 8.47 ppm to the area of the peak at 4.00 ppm. The peaks are assigned to two aromatic protons ortho to the nitrogen of **5** (8.47 ppm) and the methine proton of NIPAM (4.00 ppm). The mol fraction of **7** was calculated from the ratio of the area of the peaks at 8.15-8.36 ppm (corresponding to three aromatic protons of dansyl) to the area of the peak at 4.00 ppm of the NIPAM methine proton. The weight average molecular weight (Mw) of the polymer was determined by diffusion order spectroscopy (DOSY) NMR via a calibration curve of polystyrene standards.[1]

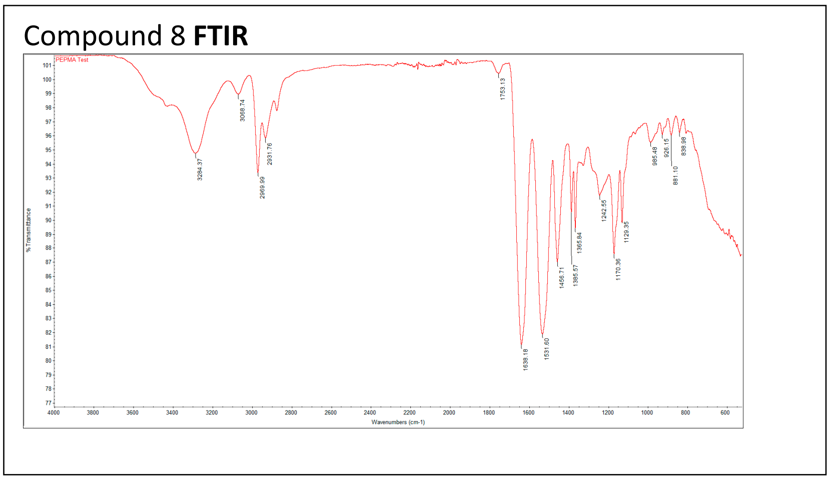

II. Fluorescence spectra

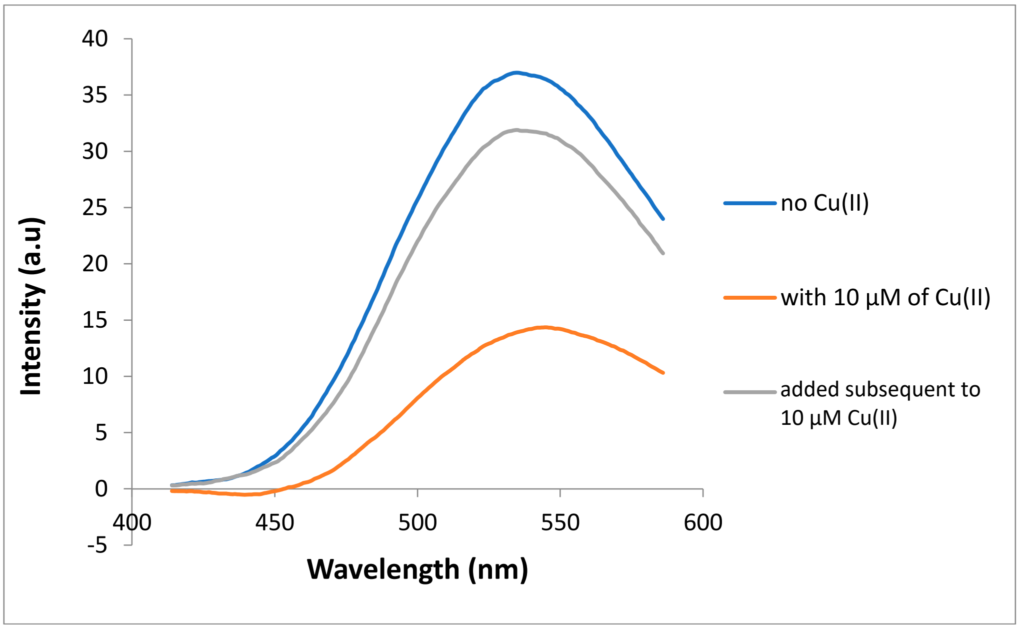

Figure S1. Addition of 10 µM Cu(II) and 20 µM EDTA solution to 0.005g/L polymer sample at 25 °C

III. Job's Plot for Cu^{2+}-PEPMA reaction

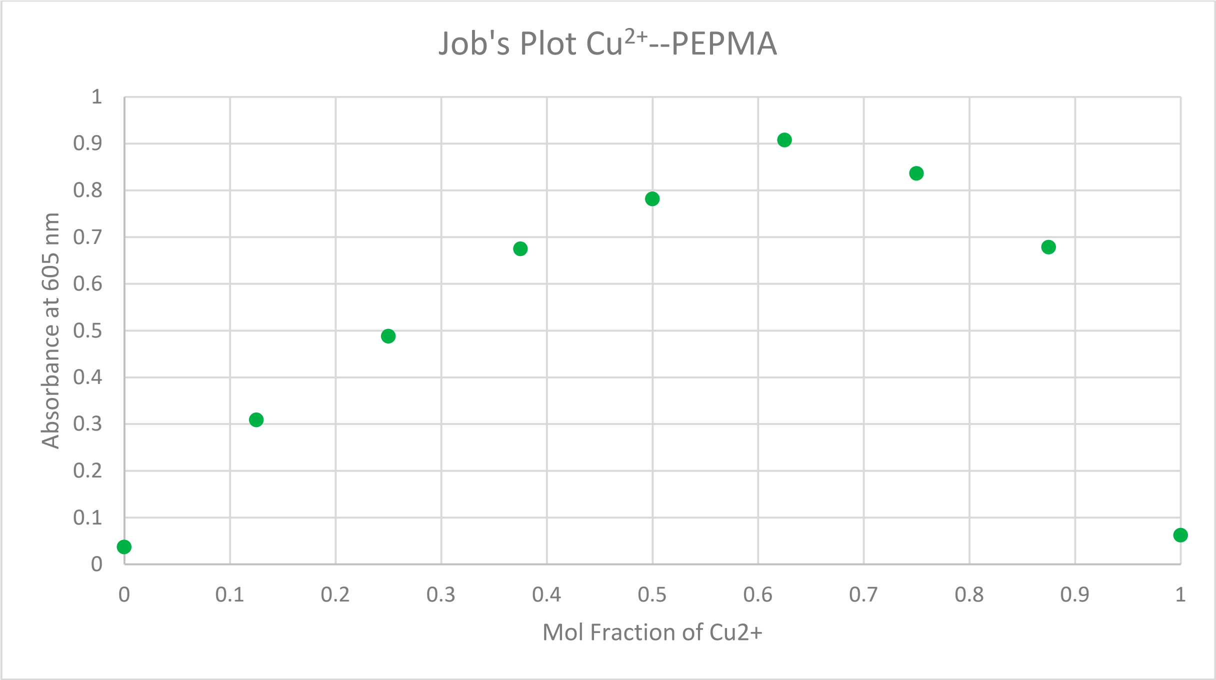

IV. Crystal structure data including CIF file of {[Cu(4)]($(ClO_4)_2$•0.5 H_2O}$_2$ ([plan23_0m])

Table 1 Crystal data and structure refinement for plan23_0m.

Identification code	plan23_0m
Empirical formula	$C_{40}H_{58}Cl_4Cu_2N_8O_{19}$
Formula weight	1223.82
Temperature/K	100.0
Crystal system	triclinic
Space group	P-1
a/Å	8.8358(5)
b/Å	14.1355(8)
c/Å	20.8894(11)
α/°	86.420(2)
β/°	85.606(2)
γ/°	79.810(2)
Volume/Å^3	2557.2(2)
Z	2
ρ_{calc}g/cm^3	1.589
μ/mm^{-1}	1.122
F(000)	1264.0
Crystal size/mm^3	0.1 × 0.05 × 0.01
Radiation	MoKα (λ = 0.71073)
2Θ range for data collection/°	3.442 to 51.506
Index ranges	-10 ≤ h ≤ 10, -17 ≤ k ≤ 17, -25 ≤ l ≤ 24
Reflections collected	40035
Independent reflections	9480 [R_{int} = 0.0433, R_{sigma} = 0.0420]
Data/restraints/parameters	9480/0/713
Goodness-of-fit on F^2	1.028
Final R indexes [I>=2σ (I)]	R_1 = 0.0557, wR_2 = 0.1231
Final R indexes [all data]	R_1 = 0.0747, wR_2 = 0.1336
Largest diff. peak/hole / e Å^{-3}	1.45/-1.00

Table 4 Bond Lengths for plan23_0m.

Atom	Atom	Length/Å	Atom	Atom	Length/Å
Cu(1)	O(4)	2.096(3)	N(7)	C(23)	1.336(6)
Cu(1)	O(5)	2.164(3)	N(8)	C(29)	1.438(6)
Cu(1)	N(1)	1.962(3)	N(8)	C(30)	1.338(5)
Cu(1)	N(2)	2.042(3)	C(1)	C(2)	1.380(6)
Cu(1)	N(3)	1.956(3)	C(2)	C(3)	1.385(6)

Cu(2)	N(10)	1.979(3)	C(3)	C(4)	1.377(6)
Cu(2)	N(6)	2.037(4)	C(4)	C(5)	1.383(6)
Cu(2)	N(7)	1.991(4)	C(5)	C(6)	1.512(6)
Cu(2)	O(24)	2.055(3)	C(7)	C(8)	1.515(6)
Cu(2)	O(25)	2.128(5)	C(8)	C(9)	1.510(5)
Cu(2)	O(25A)	2.312(9)	C(9)	C(13)	1.387(6)
Cl(1)	O(5)	1.468(3)	C(10)	C(11)	1.374(6)
Cl(1)	O(6)	1.414(3)	C(11)	C(12)	1.381(6)
Cl(1)	O(7)	1.425(3)	C(12)	C(13)	1.382(6)
Cl(1)	O(8)	1.434(3)	C(15)	C(16)	1.526(7)
Cl(3)	O(13)	1.431(4)	C(15)	C(17)	1.516(6)
Cl(3)	O(14)	1.431(3)	C(21)	C(22)	1.514(7)
Cl(3)	O(15)	1.426(3)	C(22)	C(26)	1.382(6)
Cl(3)	O(16)	1.455(3)	C(23)	C(24)	1.382(7)
Cl(4)	O(17)	1.447(3)	C(24)	C(25)	1.374(7)
Cl(4)	O(18)	1.426(3)	C(25)	C(26)	1.368(7)
Cl(4)	O(19)	1.434(3)	C(27)	C(28)	1.456(7)
Cl(4)	O(20)	1.427(3)	C(28)	C(29)	1.579(7)
C(43)	C(42)	1.523(6)	C(30)	C(31)	1.513(7)
C(43)	C(14)	1.487(6)	C(31)	C(32)	1.542(7)
C(42)	N(9)	1.453(5)	C(31)	C(33)	1.528(7)
N(9)	C(41)	1.331(5)	C(34)	C(35)	1.437(8)
O(4)	C(41)	1.244(5)	C(35)	C(36)	1.517(7)
O(10)	C(30)	1.232(5)	C(36)	C(40)	1.394(7)
N(10)	C(36)	1.311(6)	C(37)	C(38)	1.373(7)
N(10)	C(37)	1.341(6)	C(38)	C(39)	1.354(8)
N(1)	C(1)	1.339(5)	C(39)	C(40)	1.333(7)
N(1)	C(5)	1.344(5)	Cl(5)	O(26)	1.343(13)
N(2)	C(6)	1.512(6)	Cl(5)	O(25)	1.423(8)
N(2)	C(7)	1.467(6)	Cl(5)	O(27)	1.433(9)
N(2)	C(14)	1.528(5)	Cl(5)	O(28)	1.379(8)
N(3)	C(9)	1.350(5)	O(25)	O(28)	1.730(13)
N(3)	C(10)	1.345(5)	O(25A)	Cl(5A)	1.127(12)
C(41)	C(15)	1.516(6)	O(25A)	O(28A)	1.201(13)
N(6)	C(21)	1.654(7)	Cl(5A)	O(27A)	1.52(2)
N(6)	C(27)	1.494(6)	Cl(5A)	O(26A)	1.419(15)
N(6)	C(34)	1.457(6)	Cl(5A)	O(28A)	1.415(14)
N(7)	C(22)	1.345(6)			

Table 5 Bond Angles for plan23_0m.

Atom	Atom	Atom	Angle/°	Atom	Atom	Atom	Angle/°
O(4)	Cu(1)	O(5)	90.85(10)	C(22)	N(7)	Cu(2)	115.6(3)
N(1)	Cu(1)	O(4)	92.79(12)	C(23)	N(7)	Cu(2)	125.3(3)
N(1)	Cu(1)	O(5)	88.19(12)	C(23)	N(7)	C(22)	119.0(4)
N(1)	Cu(1)	N(2)	82.63(14)	C(30)	N(8)	C(29)	123.9(4)
N(2)	Cu(1)	O(4)	114.08(13)	N(1)	C(1)	C(2)	121.9(4)
N(2)	Cu(1)	O(5)	153.70(13)	C(1)	C(2)	C(3)	118.4(4)
N(3)	Cu(1)	O(4)	97.95(12)	C(4)	C(3)	C(2)	119.9(4)
N(3)	Cu(1)	O(5)	87.13(12)	C(3)	C(4)	C(5)	118.9(4)
N(3)	Cu(1)	N(1)	168.34(14)	N(1)	C(5)	C(4)	121.3(4)
N(3)	Cu(1)	N(2)	97.00(14)	N(1)	C(5)	C(6)	112.1(3)
N(10)	Cu(2)	N(6)	92.22(15)	C(4)	C(5)	C(6)	126.6(4)
N(10)	Cu(2)	N(7)	171.88(16)	C(5)	C(6)	N(2)	109.6(3)
N(10)	Cu(2)	O(24)	97.67(13)	N(2)	C(7)	C(8)	111.6(4)
N(10)	Cu(2)	O(25)	94.07(17)	C(9)	C(8)	C(7)	113.9(4)
N(10)	Cu(2)	O(25A)	89.7(2)	N(3)	C(9)	C(8)	116.9(3)
N(6)	Cu(2)	O(24)	139.90(15)	N(3)	C(9)	C(13)	120.7(4)
N(6)	Cu(2)	O(25)	114.9(2)	C(13)	C(9)	C(8)	122.4(4)
N(6)	Cu(2)	O(25A)	143.9(3)	N(3)	C(10)	C(11)	122.1(4)
N(7)	Cu(2)	N(6)	83.78(16)	C(10)	C(11)	C(12)	118.3(4)
N(7)	Cu(2)	O(24)	89.90(15)	C(11)	C(12)	C(13)	120.2(4)
N(7)	Cu(2)	O(25)	81.29(19)	C(12)	C(13)	C(9)	118.9(4)
N(7)	Cu(2)	O(25A)	89.4(2)	C(43)	C(14)	N(2)	117.1(4)
O(24)	Cu(2)	O(25)	103.1(2)	C(41)	C(15)	C(16)	110.2(4)
O(24)	Cu(2)	O(25A)	75.2(3)	C(41)	C(15)	C(17)	109.3(3)
O(6)	Cl(1)	O(5)	108.66(18)	C(17)	C(15)	C(16)	112.2(4)
O(6)	Cl(1)	O(7)	110.5(2)	C(22)	C(21)	N(6)	108.3(4)
O(6)	Cl(1)	O(8)	111.5(2)	N(7)	C(22)	C(21)	114.2(4)
O(7)	Cl(1)	O(5)	108.76(19)	N(7)	C(22)	C(26)	121.2(4)
O(7)	Cl(1)	O(8)	108.74(19)	C(26)	C(22)	C(21)	124.6(4)
O(8)	Cl(1)	O(5)	108.63(16)	N(7)	C(23)	C(24)	122.2(4)
O(13)	Cl(3)	O(16)	108.3(2)	C(25)	C(24)	C(23)	118.6(5)
O(14)	Cl(3)	O(13)	110.9(2)	C(26)	C(25)	C(24)	119.5(4)
O(14)	Cl(3)	O(16)	108.9(2)	C(25)	C(26)	C(22)	119.4(4)
O(15)	Cl(3)	O(13)	110.2(2)	C(28)	C(27)	N(6)	109.9(4)
O(15)	Cl(3)	O(14)	111.0(2)	C(27)	C(28)	C(29)	113.6(4)
O(15)	Cl(3)	O(16)	107.4(2)	N(8)	C(29)	C(28)	112.0(4)
O(18)	Cl(4)	O(17)	109.7(2)	O(10)	C(30)	N(8)	120.7(4)
O(18)	Cl(4)	O(19)	110.2(2)	O(10)	C(30)	C(31)	122.1(4)
O(18)	Cl(4)	O(20)	110.0(2)	N(8)	C(30)	C(31)	117.2(4)
O(19)	Cl(4)	O(17)	108.5(2)	C(30)	C(31)	C(32)	108.6(4)
O(20)	Cl(4)	O(17)	109.20(19)	C(30)	C(31)	C(33)	113.5(4)

O(20)	Cl(4)	O(19)	109.3(2)	C(33)	C(31)	C(32)	111.0(4)
C(14)	C(43)	C(42)	117.5(4)	C(35)	C(34)	N(6)	113.9(5)
N(9)	C(42)	C(43)	114.1(3)	C(34)	C(35)	C(36)	104.7(5)
C(41)	N(9)	C(42)	125.1(4)	N(10)	C(36)	C(35)	118.9(4)
C(41)	O(4)	Cu(1)	142.3(3)	N(10)	C(36)	C(40)	122.1(5)
Cl(1)	O(5)	Cu(1)	111.60(14)	C(40)	C(36)	C(35)	118.5(5)
C(36)	N(10)	Cu(2)	119.0(3)	N(10)	C(37)	C(38)	122.9(5)
C(36)	N(10)	C(37)	116.8(4)	C(39)	C(38)	C(37)	119.3(5)
C(37)	N(10)	Cu(2)	124.1(3)	C(40)	C(39)	C(38)	118.3(5)
C(1)	N(1)	Cu(1)	125.1(3)	C(39)	C(40)	C(36)	120.4(5)
C(1)	N(1)	C(5)	119.7(3)	O(26)	Cl(5)	O(25)	118.5(6)
C(5)	N(1)	Cu(1)	115.1(3)	O(26)	Cl(5)	O(27)	116.1(6)
C(6)	N(2)	Cu(1)	101.5(2)	O(26)	Cl(5)	O(28)	115.5(8)
C(6)	N(2)	C(14)	106.2(3)	O(25)	Cl(5)	O(27)	117.1(6)
C(7)	N(2)	Cu(1)	115.1(3)	O(28)	Cl(5)	O(25)	76.3(6)
C(7)	N(2)	C(6)	109.6(3)	O(28)	Cl(5)	O(27)	105.8(6)
C(7)	N(2)	C(14)	113.1(3)	Cl(5)	O(25)	Cu(2)	139.6(4)
C(14)	N(2)	Cu(1)	110.3(3)	Cl(5)	O(25)	O(28)	50.7(4)
C(9)	N(3)	Cu(1)	120.7(3)	O(28)	O(25)	Cu(2)	129.4(4)
C(10)	N(3)	Cu(1)	119.2(3)	Cl(5)	O(28)	O(25)	53.0(5)
C(10)	N(3)	C(9)	119.8(3)	Cl(5A)	O(25A)	Cu(2)	133.7(7)
N(9)	C(41)	C(15)	117.4(4)	Cl(5A)	O(25A)	O(28A)	74.8(9)
O(4)	C(41)	N(9)	123.4(4)	O(28A)	O(25A)	Cu(2)	138.0(9)
O(4)	C(41)	C(15)	119.2(4)	O(25A)	Cl(5A)	O(27A)	116.1(11)
C(21)	N(6)	Cu(2)	100.9(2)	O(25A)	Cl(5A)	O(26A)	139.6(11)
C(27)	N(6)	Cu(2)	113.5(3)	O(25A)	Cl(5A)	O(28A)	55.0(7)
C(27)	N(6)	C(21)	106.7(4)	O(26A)	Cl(5A)	O(27A)	104.0(12)
C(34)	N(6)	Cu(2)	115.2(4)	O(28A)	Cl(5A)	O(27A)	109.5(13)
C(34)	N(6)	C(21)	106.3(4)	O(28A)	Cl(5A)	O(26A)	108.2(12)
C(34)	N(6)	C(27)	112.9(4)	O(25A)	O(28A)	Cl(5A)	50.2(7)

Table 6 Torsion Angles for plan23_0m.

A	B	C	D	Angle/°	A	B	C	D	Angle/°
Cu(1)	O(4)	C(41)	N(9)	-24.0(7)	C(2)	C(3)	C(4)	C(5)	0.7(6)
Cu(1)	O(4)	C(41)	C(15)	156.4(3)	C(3)	C(4)	C(5)	N(1)	-0.6(6)
Cu(1)	N(1)	C(1)	C(2)	179.7(3)	C(3)	C(4)	C(5)	C(6)	177.7(4)
Cu(1)	N(1)	C(5)	C(4)	-179.4(3)	C(4)	C(5)	C(6)	N(2)	148.4(4)
Cu(1)	N(1)	C(5)	C(6)	2.1(4)	C(5)	N(1)	C(1)	C(2)	0.1(6)

Cu(1)	N(2)	C(6)	C(5)	45.1(4)	C(6)	N(2)	C(7)	C(8)	-82.5(4)
Cu(1)	N(2)	C(7)	C(8)	31.1(4)	C(6)	N(2)	C(14)	C(43)	-166.7(4)
Cu(1)	N(2)	C(14)	C(43)	84.1(4)	C(7)	N(2)	C(6)	C(5)	167.2(3)
Cu(1)	N(3)	C(9)	C(8)	5.0(5)	C(7)	N(2)	C(14)	C(43)	-46.5(5)
Cu(1)	N(3)	C(9)	C(13)	-173.8(3)	C(7)	C(8)	C(9)	N(3)	56.2(5)
Cu(1)	N(3)	C(10)	C(11)	173.2(3)	C(7)	C(8)	C(9)	C(13)	-125.1(4)
Cu(2)	N(10)	C(36)	C(35)	4.8(7)	C(8)	C(9)	C(13)	C(12)	-177.9(4)
Cu(2)	N(10)	C(36)	C(40)	176.1(5)	C(9)	N(3)	C(10)	C(11)	-0.8(6)
Cu(2)	N(10)	C(37)	C(38)	-175.1(5)	C(10)	N(3)	C(9)	C(8)	179.0(4)
Cu(2)	N(6)	C(21)	C(22)	-41.6(4)	C(10)	N(3)	C(9)	C(13)	0.2(6)
Cu(2)	N(6)	C(27)	C(28)	-58.0(5)	C(10)	C(11)	C(12)	C(13)	0.6(7)
Cu(2)	N(6)	C(34)	C(35)	-25.8(6)	C(11)	C(12)	C(13)	C(9)	-1.3(7)
Cu(2)	N(7)	C(22)	C(21)	2.0(6)	C(14)	C(43)	C(42)	N(9)	-70.8(5)
Cu(2)	N(7)	C(22)	C(26)	-177.0(4)	C(14)	N(2)	C(6)	C(5)	-70.3(4)
Cu(2)	N(7)	C(23)	C(24)	175.4(4)	C(14)	N(2)	C(7)	C(8)	159.2(3)
Cu(2)	O(25)	O(28)	Cl(5)	-127.0(6)	C(21)	N(6)	C(27)	C(28)	-168.3(4)
Cu(2)	O(25A)	Cl(5A)	O(27A)	120.0(12)	C(21)	N(6)	C(34)	C(35)	85.0(6)
Cu(2)	O(25A)	Cl(5A)	O(26A)	-67(2)	C(21)	C(22)	C(26)	C(25)	-177.8(5)
Cu(2)	O(25A)	Cl(5A)	O(28A)	-143.7(13)	C(22)	N(7)	C(23)	C(24)	-0.4(9)
Cu(2)	O(25A)	O(28A)	Cl(5A)	140.3(12)	C(23)	N(7)	C(22)	C(21)	178.2(5)
C(43)	C(42)	N(9)	C(41)	99.2(5)	C(23)	N(7)	C(22)	C(26)	-0.8(8)
C(42)	C(43)	C(14)	N(2)	-61.0(5)	C(23)	C(24)	C(25)	C(26)	-0.9(8)
C(42)	N(9)	C(41)	O(4)	-6.0(6)	C(24)	C(25)	C(26)	C(22)	-0.2(8)
C(42)	N(9)	C(41)	C(15)	173.5(4)	C(27)	N(6)	C(21)	C(22)	77.2(5)
N(9)	C(41)	C(15)	C(16)	115.0(4)	C(27)	N(6)	C(34)	C(35)	-158.3(5)
N(9)	C(41)	C(15)	C(17)	-121.2(4)	C(27)	C(28)	C(29)	N(8)	-67.1(6)
O(4)	C(41)	C(15)	C(16)	-65.4(5)	C(29)	N(8)	C(30)	O(10)	7.6(7)
O(4)	C(41)	C(15)	C(17)	58.4(5)	C(29)	N(8)	C(30)	C(31)	-169.2(4)
O(6)	Cl(1)	O(5)	Cu(1)	-137.2(2)	C(30)	N(8)	C(29)	C(28)	95.1(5)
O(7)	Cl(1)	O(5)	Cu(1)	102.45(19)	C(34)	N(6)	C(21)	C(22)	-162.1(4)
O(8)	Cl(1)	O(5)	Cu(1)	-15.8(2)	C(34)	N(6)	C(27)	C(28)	75.3(6)
O(10)	C(30)	C(31)	C(32)	-96.4(5)	C(34)	C(35)	C(36)	N(10)	-68.3(6)
O(10)	C(30)	C(31)	C(33)	139.8(5)	C(34)	C(35)	C(36)	C(40)	120.1(6)
N(10)	C(36)	C(40)	C(39)	2.7(10)	C(35)	C(36)	C(40)	C(39)	173.9(6)
N(10)	C(37)	C(38)	C(39)	-4.3(11)	C(36)	N(10)	C(37)	C(38)	4.6(10)
N(1)	C(1)	C(2)	C(3)	0.0(6)	C(37)	N(10)	C(36)	C(35)	-174.9(6)
N(1)	C(5)	C(6)	N(2)	-33.2(5)	C(37)	N(10)	C(36)	C(40)	-3.7(8)
N(2)	C(7)	C(8)	C(9)	-76.3(5)	C(37)	C(38)	C(39)	C(40)	3.0(10)
N(3)	C(9)	C(13)	C(12)	0.8(6)	C(38)	C(39)	C(40)	C(36)	-2.2(9)
N(3)	C(10)	C(11)	C(12)	0.4(6)	O(26)	Cl(5)	O(25)	Cu(2)	-140.5(9)
N(6)	C(21)	C(22)	N(7)	27.6(6)	O(26)	Cl(5)	O(25)	O(28)	111.8(9)
N(6)	C(21)	C(22)	C(26)	-153.5(5)	O(26)	Cl(5)	O(28)	O(25)	-115.4(7)

N(6)	C(27)	C(28)	C(29)	179.7(4)	O(27)	Cl(5)	O(25)	Cu(2)	6.8(9)
N(6)	C(34)	C(35)	C(36)	78.0(6)	O(27)	Cl(5)	O(25)	O(28)	-101.0(6)
N(7)	C(22)	C(26)	C(25)	1.1(8)	O(27)	Cl(5)	O(28)	O(25)	114.7(6)
N(7)	C(23)	C(24)	C(25)	1.2(9)	O(28)	Cl(5)	O(25)	Cu(2)	107.7(7)
N(8)	C(30)	C(31)	C(32)	80.3(5)	O(27A)	Cl(5A)	O(28A)	O(25A)	108.8(11)
N(8)	C(30)	C(31)	C(33)	-43.5(5)	O(26A)	Cl(5A)	O(28A)	O(25A)	-138.4(12)
C(1)	N(1)	C(5)	C(4)	0.2(6)	O(28A)	O(25A)	Cl(5A)	O(27A)	-96.3(14)
C(1)	N(1)	C(5)	C(6)	-178.3(4)	O(28A)	O(25A)	Cl(5A)	O(26A)	76(2)
C(1)	C(2)	C(3)	C(4)	-0.4(6)					

Supplemental Reference

1. Li, W.; Chung, H.; Daeffler, C.; Johnson, J. A.; Grubbs, R. H., Application of 1H DOSY for Facile Measurement of Polymer Molecular Weights. *Macromolecules* **2012,** *45* (24), 9595-9603.
